# Synbiotics as a Microbiome-Based Strategy in Colorectal Cancer

**DOI:** 10.3390/nu18101591

**Published:** 2026-05-16

**Authors:** Lucia Maria Procopciuc, Adrina Corina Hangan, Roxana Liana Lucaciu

**Affiliations:** 1 Department of Medical Biochemistry, “Iuliu Hațieganu” University of Medicine and Pharmacy, 400349 Cluj-Napoca, Romania; lprocopciuc@umfcluj.ro; 2 Department of Inorganic Chemistry, Faculty of Pharmacy, “Iuliu Hațieganu” University of Medicine and Pharmacy, 400012 Cluj-Napoca, Romania; 3 Department of Pharmaceutical Biochemistry and Clinical Laboratory, Faculty of Pharmacy, “Iuliu Hațieganu” University of Medicine and Pharmacy, 400349 Cluj-Napoca, Romania; liana.lucaciu@umfcluj.ro

**Keywords:** colorectal cancer, synbiotics, microbiome

## Abstract

Colorectal cancer (CRC) is a multifactorial disease arising from dynamic interactions between gut microbiota, inflammatory processes, metabolic reprogramming, and dysregulated host signaling pathways. Increasing evidence highlights the potential of synbiotics—combinations of probiotics and prebiotics—as promising modulators of these processes. This review explores the mechanisms by which synbiotics influence CRC development and progression, integrating data from preclinical and clinical studies. Synbiotics exert beneficial effects by restoring microbial balance, enhancing the production of short-chain fatty acids (SCFAs), strengthening intestinal barrier integrity, and reducing chronic inflammation and oxidative stress. These functional changes converge on key molecular pathways, including Wnt/β-catenin, NF-κB, and PI3K/Akt/mTOR, which regulate tumor cell proliferation, survival, and immune responses. Preclinical studies consistently demonstrate anti-tumor effects, including reduced tumor growth, increased apoptosis, and modulation of the tumor microenvironment. Clinical evidence suggests that synbiotics may improve postoperative outcomes, reduce chemotherapy-related toxicity, and positively influence microbiome composition, although results remain heterogeneous. Emerging approaches focusing on microbiome profiling and personalized synbiotic interventions offer new opportunities for precision medicine in CRC. Overall, synbiotics represent a promising adjunctive strategy in colorectal cancer management, with potential to enhance therapeutic efficacy and improve patient outcomes. Further large-scale clinical studies are needed to validate their long-term benefits and establish standardized treatment protocols.

## 1. Introduction

Colorectal cancer (CRC) represents a major global public health challenge due to its high incidence, significant mortality risk, and significant socio-economic impact. According to recent estimates, CRC is the third most commonly diagnosed cancer and the second leading cause of cancer-related death worldwide, accounting for nearly 10% of all cancer cases and deaths. In 2022, approximately 1.9 million new cases and over 900,000 deaths were reported globally, highlighting the considerable burden of this disease [1].

The global distribution of CRC shows marked geographical disparities. Incidence rates are highest in high-income regions such as Europe, North America, and Australia, whereas mortality rates tend to be disproportionately higher in low- and middle-income countries, reflecting inequalities in access to screening, early diagnosis, and treatment [2,3]. These variations are strongly associated with differences in lifestyle factors, including diet, physical inactivity, obesity, alcohol consumption, and smoking, which are more prevalent in industrialized societies [4].

Over recent decades, the global burden of CRC has continued to increase, driven by population growth, aging, and the widespread adoption of Westernized lifestyles [5]. Projections indicate that the number of new CRC cases may exceed 2.5 million annually by 2040, emphasizing the urgent need for effective prevention and control [6]. Furthermore, an alarming rise in early-onset CRC (diagnosed before the age of 50) has been observed worldwide, suggesting the involvement of additional environmental and possibly microbiome-related risk factors [7].

Despite advances in screening and treatment, CRC remains associated with considerable morbidity and mortality, particularly when diagnosed at advanced stages. Early detection through screening programs has been shown to significantly reduce both incidence and mortality; however, implementation and participation rates remain suboptimal in many regions [4]. Consequently, strengthening global prevention strategies, improving access to screening, and addressing health disparities are essential to reduce the burden of colorectal cancer worldwide.

Despite significant advances in the management of CRC, current therapeutic strategies, including surgery, chemotherapy, and immunotherapy, are still associated with important limitations that affect long-term outcomes and patient survival.

Surgery remains the main treatment for early-stage and locally advanced CRC, with curative potential in resectable disease. However, many patients are diagnosed at advanced stages when surgery is no longer curative [8]. Even after resection, recurrence remains common due to micrometastases or residual disease, while postoperative complications may reduce quality of life [9]. Moreover, surgery alone is insufficient for systemic disease, requiring adjuvant therapies. Chemotherapy is widely used in both adjuvant and metastatic CRC, improving survival outcomes in many patients. However, its efficacy is limited by drug resistance, tumor heterogeneity, and systemic toxicity [10]. Common adverse effects, including myelosuppression, gastrointestinal toxicity, neuropathy, and fatigue, negatively affect quality of life and treatment efficiency [11]. In advanced CRC, chemotherapy is often palliative rather than curative.

Immunotherapy has emerged as a promising treatment modality, particularly immune checkpoint inhibitors targeting PD-1/PD-L1 pathways. While these therapies have shown remarkable efficacy in patients with microsatellite instability-high (MSI-H) or mismatch repair-deficient (dMMR) tumors, their benefit is limited to a small subset of CRC patients [12]. The majority of CRC cases are microsatellite stable (MSS), characterized by an immunologically “cold” tumor microenvironment that does not respond effectively to immunotherapy.

Additionally, resistance to immunotherapy remains a major challenge. Mechanisms include immune evasion, suppressive tumor microenvironment, and inadequate T-cell infiltration [13]. Even in responsive patients, not all achieve durable responses, and immune-related adverse events (e.g., colitis, dermatitis, endocrinopathies) can limit treatment use. Consequently, immunotherapy alone is insufficient for most CRC patients and often requires combination strategies with chemotherapy or targeted therapies [14].

Across all treatment modalities, several overarching limitations persist: tumor heterogeneity, leading to variable treatment responses; late-stage diagnosis, reducing curative potential; development of resistance mechanisms; treatment-related toxicity and reduced quality of life; and limited efficacy in metastatic disease, where CRC remains incurable in approximately 50% of cases [14]. These limitations highlight the need for novel therapeutic approaches, including precision medicine, targeted therapies, and combination treatment strategies aimed at overcoming resistance and improving patient outcomes.

In this context, the gut microbiota, defined as the community of microorganisms inhabiting the gastrointestinal tract, and the gut microbiome, referring to their collective genetic material, have emerged as critical factors in CRC pathogenesis and treatment response. The intestinal microbiota plays a key role in maintaining homeostasis, regulating immune responses, and influencing metabolic processes. Dysbiosis has been strongly associated with colorectal carcinogenesis through mechanisms including chronic inflammation, production of genotoxic metabolites, and disruption of epithelial barrier integrity [15,16]. Specific microbial species, such as *Fusobacterium nucleatum* and colibactin-producing *Escherichia coli*, have been shown to promote tumor initiation and progression by inducing DNA damage and modulating host immune responses [17,18].

The growing understanding of microbiome–host interactions has provided a strong rationale for microbiome-targeted therapeutic strategies. Unlike genetic alterations, the gut microbiome is modifiable, making it an attractive target for intervention. Modulation of the microbiota through dietary interventions, synbiotics, antibiotics, or fecal microbiota transplantation (FMT) has the potential to restore microbial balance, reduce inflammation, and enhance anti-tumor immunity [16,19]. Furthermore, the microbiome has been shown to influence the efficacy and toxicity of chemotherapy and immunotherapy, suggesting that microbiome-based interventions could improve treatment outcomes and overcome resistance mechanisms [20].

Synbiotics, defined as combinations of probiotics and prebiotics, have emerged as a promising strategy for modulating the gut microbiome in CRC. They promote the growth and activity of beneficial microorganisms and increase SCFA production, particularly butyrate, which exerts anti-inflammatory and anti-tumor effects [21,22]. In addition, synbiotics may reduce carcinogenic metabolites and inhibit pathogenic bacteria involved in CRC development [23].

Preclinical and clinical studies suggest that synbiotics may play a role in both CRC prevention and as an adjuvant to conventional therapies. They have been associated with reduced inflammation, improved intestinal barrier function, and modulation of immune responses, as well as improved postoperative outcomes and reduced treatment-related toxicity [24]. However, despite promising results, their clinical application remains limited by heterogeneity in study design, variability in microbiome composition among individuals, and the need for standardized therapeutic protocols [21].

The main objective of this study is to evaluate the role of the gut microbiome in CRC development, with a particular focus on the administration of synbiotics as a complementary strategy for prevention and treatment. Additionally, the study aims to assess the limitations of current therapies and explore how synbiotic supplementation may enhance therapeutic efficacy, modulate treatment response, and support personalized CRC management.

This manuscript is presented as a narrative review that aims to provide a comprehensive and integrative overview of current evidence regarding the role of synbiotics in colorectal cancer. Unlike systematic reviews or meta-analyses, this narrative review focuses on synthesizing mechanistic, preclinical, and clinical evidence to highlight the complex interactions between gut microbiota, synbiotic-derived metabolites, inflammatory responses, and intracellular oncogenic signaling pathways, including Wnt/β-catenin, NF-κB, and PI3K/Akt/mTOR. In addition, the review discusses emerging perspectives related to precision medicine and personalized microbiome-targeted interventions in CRC.

## 2. The Gut Microbiota and Colorectal Cancer

### 2.1. Composition and Function of the Gut Microbiota

The gut microbiota represents a highly complex and dynamic ecosystem composed of trillions of microorganisms, as well as a structured microbial ecosystem composed of multiple domains of life, each contributing uniquely to intestinal homeostasis and disease processes.

The gut microbiota includes bacteria, archaea, viruses, and fungi, that reside predominantly in the large intestine. It is estimated that the human gut contains over 10^13^–10^14^ microbial cells, with a gene content vastly exceeding that of the human genome, collectively referred to as the microbiome [25,26]. There are several dominant phyla playing essential roles in maintaining intestinal homeostasis. The dominant bacterial phyla in the healthy gut are *Firmicutes* and *Bacteroidetes*, followed by *Actinobacteria*, *Proteobacteria*, and *Verrucomicrobia*, although their relative abundance varies between individuals [27].

The composition of the gut microbiota is influenced by multiple factors, including genetics, diet, age, environment, medication use (especially antibiotics), and lifestyle [28]. A balanced microbiota is characterized by high microbial diversity and stability, which are essential for maintaining intestinal homeostasis.

Its composition is not only taxonomically diverse but also functionally specialized, varying across individuals and along different regions of the gastrointestinal tract.

*Firmicutes*, including genera such as *Clostridium*, *Lactobacillus*, and *Faecalibacterium*, are primarily involved in the production of SCFAs, particularly butyrate, which is crucial for colonocyte energy metabolism and has anti-inflammatory effects. Additionally, the microbiota is involved in bile acid metabolism, vitamin synthesis (e.g., vitamin K and some B vitamins), and regulation of host energy balance [29]. *Bacteroidetes*, represented by genera such as *Bacteroides* and *Prevotella*, are mainly associated with the breakdown of complex carbohydrates and fermentation processes. *Actinobacteria*, especially *Bifidobacterium*, contribute to gut barrier integrity and play an important role in immune modulation. Microbial components and metabolites can influence the differentiation of regulatory T cells (Tregs) and the production of cytokines, thereby modulating inflammation [30]. In contrast, *Proteobacteria*, including genera such as *Escherichia* and *Enterobacter*, are typically present in low abundance in healthy individuals but tend to increase under dysbiotic conditions, often reflecting microbial imbalance. Finally, *Verrucomicrobia*, particularly *Akkermansia muciniphila*, are involved in mucin degradation and are closely linked to the maintenance of gut barrier function, preventing the translocation of harmful pathogens and toxins [31]. In healthy individuals, *Firmicutes* and *Bacteroidetes* together account for approximately 90% of the gut microbiota [32,33]. The ratio between these phyla is often used as an indicator of microbial balance, although its interpretation remains complex.

Although less abundant than bacteria, other microbial components also contribute significantly to the gut ecosystem. Archaea such as *Methanobrevibacter smithii* are involved in gas metabolism and microbial interactions [34], while the gut virome regulates bacterial populations and influences microbial diversity and gene transfer [35]. In addition, fungi forming the mycobiome, including *Candida*, *Saccharomyces*, and *Malassezia*, interact with both bacterial communities and the host immune system, and have been associated with inflammatory and neoplastic conditions [36].

The composition of the gut microbiota varies along the gastrointestinal tract according to differences in pH, oxygen levels, nutrient availability, and intestinal transit time. Microbial density is low in the stomach and proximal small intestine, where facultative anaerobes predominate, whereas the colon harbors the highest microbial density—up to 10^12^ cells per gram of content—and is mainly populated by obligate anaerobes such as *Bacteroides* and *Clostridium* species [33]. This spatial heterogeneity contributes to the functional specialization of microbial communities and their role in diseases such as CRC.

Beyond its taxonomic composition, the gut microbiota is characterized by extensive functional redundancy, meaning that different microbial species are capable of performing similar metabolic functions. The collective genome of the microbiota—referred to as the microbiome—contains millions of genes involved in a wide range of essential biological processes, including carbohydrate metabolism such as dietary fiber fermentation, amino acid synthesis, xenobiotic metabolism, and bile acid transformation. This remarkable functional capacity enables the microbiome to adapt to environmental changes and maintain host homeostasis, even in the presence of fluctuations in microbial composition [37].

The composition of the gut microbiota is shaped by a variety of interconnected factors that influence microbial diversity and stability. Among these, diet is one of the most significant determinants, as high-fiber diets promote the growth of beneficial bacteria, whereas high-fat and low-fiber diets are associated with dysbiosis. The use of antibiotics can profoundly disrupt microbial balance, often leading to a reduction in diversity and long-term alterations in microbial communities [28]. Age also plays an important role, with the microbiota undergoing dynamic changes from infancy through adulthood and into old age. In addition, host genetics contribute to shaping microbial colonization patterns and modulating immune interactions with the microbiota. Finally, lifestyle and environmental factors—including stress, hygiene practices, and geographic location—further influence the structure and function of the gut microbiota [38].

### 2.2. Dysbiosis in Colorectal Cancer

Dysbiosis refers to a disruption in the composition, diversity, and functional capacity of the gut microbiota, resulting in an imbalance between beneficial commensal microorganisms and potentially harmful species. In the context of CRC, dysbiosis is characterized not only by taxonomic alterations but also by profound metabolic and functional changes that influence host physiology and disease progression [39,40].

Under physiological conditions, the gut microbiota exists in a synbiotic relationship with the host, contributing to nutrient metabolism, maintenance of intestinal barrier integrity, and immune system regulation. However, in dysbiosis, this balance is disrupted, leading to a reduction in microbial diversity (alpha diversity) and altered community structure (beta diversity), both of which have been consistently observed in CRC patients [40,41].

Metagenomic, metatranscriptomic, and metabolomic studies have revealed that CRC-associated microbiota exhibit distinct microbial signatures compared to healthy individuals. These include enrichment of pro-inflammatory and pro-carcinogenic bacteria, such as *Fusobacterium nucleatum* and enterotoxigenic *Bacteroides fragilis*, alongside depletion of protective commensals [41,42].

A key feature of CRC-associated dysbiosis is the depletion of beneficial bacteria, particularly those responsible for the production of SCFAs, especially butyrate. Functionally, dysbiosis is associated with altered microbial metabolic pathways, including increased production of harmful metabolites (e.g., secondary bile acids, hydrogen sulfide) and decreased synthesis of beneficial compounds such as SCFAs [39]. These functional shifts contribute directly to epithelial damage, inflammation, and carcinogenesis.

Importantly, the microbial changes observed in CRC are spatially heterogeneous, with tumor-associated microbiota differing from those found in adjacent normal mucosa and fecal samples, suggesting a localized tumor–microbiome interaction [42].

Prominent butyrate-producing species include *Faecalibacterium prausnitzii*, *Roseburia* spp., and *Eubacterium rectale* [39,43]. Butyrate, a key short-chain fatty acid produced through microbial fermentation of dietary fibers, plays a central role in maintaining colonic homeostasis through multiple complementary mechanisms. It serves as the primary energy source for colonocytes, supporting cellular metabolism, differentiation, and normal epithelial turnover, thereby sustaining the functional integrity of the colonic mucosa [43].

In addition to its metabolic role, butyrate is essential for maintaining epithelial barrier integrity. It enhances the expression and assembly of tight junction proteins, such as claudins and occludins, which are critical for preserving mucosal barrier function and preventing the translocation of luminal bacteria and their associated toxins into underlying tissues [39]. This barrier-supporting effect is crucial in limiting chronic inflammation and maintaining intestinal homeostasis.

Furthermore, butyrate demonstrates significant anti-tumor activity. It can induce apoptosis and cell cycle arrest in CRC cells by regulating gene expression through epigenetic mechanisms and activating intrinsic apoptotic pathways. This dual role, supporting normal epithelial cells while inhibiting malignant transformation, highlights the importance of butyrate in protecting against colorectal carcinogenesis [39,43]. Collectively, these functions position butyrate as a critical mediator of gut health, linking microbial metabolism to epithelial integrity, immune regulation, and cancer prevention.

The depletion of these beneficial bacteria results in reduced SCFA production, compromising epithelial integrity and increasing intestinal permeability (“leaky gut”). This allows microbial products such as lipopolysaccharides (LPS) to penetrate the mucosa, triggering chronic inflammation and promoting tumorigenesis [41].

Furthermore, reduced butyrate levels alter the metabolic landscape of colonocytes, shifting them toward glycolytic metabolism (Warburg effect), which favors tumor growth and progression [43].

### 2.3. Key Microbial Players in Carcinogenesis

CRC-associated dysbiosis is also characterized by an increased abundance of opportunistic and pathogenic bacteria that contribute to carcinogenesis through multiple mechanisms [40,42].

*Fusobacterium nucleatum* is one of the most consistently enriched bacterial species in CRC tissues and has been strongly associated with tumor progression, metastasis, and poor clinical outcomes [42]. Its pro-tumorigenic effects are mediated through direct interactions with epithelial cells, activation of oncogenic *Fusobacterium* signaling pathways, and modulation of the host immune response.

A central mechanism by which *Fusobacterium nucleatum* promotes carcinogenesis involves its ability to adhere to and invade colorectal epithelial cells. This process is primarily mediated by the bacterial adhesin FadA, which binds to E-cadherin on the surface of host epithelial cells. The binding of FadA disrupts adherens junctions and triggers intracellular signaling cascades, most notably the activation of the β-catenin pathway.

Activation of β-catenin results in its translocation to the nucleus, where it acts as a transcriptional co-activator for genes involved in cell proliferation and survival, including *c-Myc* and *cyclin D1*. This leads to increased epithelial cell proliferation, reduced apoptosis, and enhanced tumor growth. In addition, *Fusobacterium nucleatum* promotes inflammatory signaling, further contributing to a tumor-supportive microenvironment [44].

Beyond its direct effects on epithelial signaling, *Fusobacterium nucleatum* plays a critical role in immune modulation within the tumor microenvironment. The bacterium expresses the Fap2 protein, which interacts with the inhibitory receptor TIGIT (T cell immunoreceptor with Ig and ITIM domains) expressed on natural killer (NK) cells and cytotoxic T lymphocytes. This interaction suppresses immune cell cytotoxicity and enables tumor cells to evade immune surveillance [45].

Furthermore, *Fusobacterium nucleatum* contributes to immune evasion by promoting an immunosuppressive microenvironment, characterized by reduced anti-tumor immune responses and increased tumor tolerance.

In addition to immune modulation, *Fusobacterium nucleatum* activates innate immune signaling pathways, particularly through Toll-like receptor 4 (TLR4), leading to activation of NF-κB signaling. Activation of NF-κB leads to transcription of pro-inflammatory genes, including cytokines, as well as enzymes involved in inflammatory responses, thereby sustaining a chronic inflammatory state that supports tumor development [6].

Collectively, these findings demonstrate that *Fusobacterium nucleatum* acts as a key driver of colorectal carcinogenesis by integrating adhesion-mediated signaling, inflammatory pathway activation, and immune suppression, thereby creating a microenvironment that supports tumor development and progression [44,45].

*Enterotoxigenic Bacteroides fragilis* (ETBF) represents a key microbial driver in colorectal carcinogenesis due to its ability to produce a potent virulence factor known as Bacteroides fragilis toxin (BFT), also referred to as fragilysin. BFT is a zinc-dependent metalloprotease that specifically targets epithelial cell junctional proteins, thereby disrupting intestinal barrier integrity and promoting a pro-inflammatory microenvironment [46,47]. At the epithelial level, BFT cleaves E-cadherin, a critical component of adherens junctions, leading to loss of cell–cell adhesion and increased intestinal permeability. This disruption facilitates the translocation of luminal bacteria and microbial products into the mucosa, triggering sustained immune activation and chronic inflammation [46].

In addition to its structural effects, BFT activates multiple oncogenic signaling pathways. One of the most important is the NF-κB pathway, which is rapidly induced following epithelial exposure to ETBF [47,48].

Furthermore, ETBF has been shown to activate the Signal Transducer and Activator of Transcription 3 (STAT3) signaling pathway, which plays a crucial role in cell proliferation, survival, and tumor progression. Persistent STAT3 activation enhances the expression of genes involved in anti-apoptotic signaling and cell cycle progression, contributing to uncontrolled epithelial cell growth [47].

A distinctive feature of ETBF-induced carcinogenesis is its ability to promote a Th17-mediated immune response. ETBF colonization stimulates the production of interleukin-17 (IL-17) through activation of Th17 cells, which has been strongly implicated in tumor-promoting inflammation. IL-17 further amplifies inflammatory signaling, recruits immune cells, and enhances tumor cell proliferation, creating a feedback loop that sustains chronic inflammation and tumor progression [48,49].

In addition, ETBF-induced inflammation is associated with increased production of reactive oxygen species (ROS), which can cause oxidative DNA damage and genomic instability in colonocytes. Over time, this contributes to the accumulation of mutations in key oncogenes and tumor suppressor genes, facilitating tumor initiation and progression [47].

Experimental models have demonstrated that ETBF colonization can directly induce colonic tumor formation, particularly in genetically susceptible hosts, supporting a causal role in CRC development rather than a mere association.

Overall, ETBF contributes to colorectal carcinogenesis through a multifaceted mechanism involving epithelial barrier disruption, activation of oncogenic and inflammatory signaling pathways, immune modulation, and induction of genomic instability, positioning it as a critical microbial factor in CRC pathogenesis [46,47,48,49].

Certain strains of *Escherichia coli* harbor the polyketide synthase (pks) genomic island, a gene cluster responsible for the biosynthesis of colibactin, a highly reactive genotoxic compound that plays a central role in colorectal carcinogenesis. These pks^+^ strains have been consistently detected in higher abundance within colorectal tumor tissues and precancerous lesions, supporting their involvement in both tumor initiation and progression [50,51].

Colibactin exerts its carcinogenic effect primarily through direct interaction with host DNA. Mechanistically, it acts as an alkylating agent that forms interstrand DNA crosslinks (ICLs), which interfere with DNA replication and transcription. These lesions can stall replication forks and ultimately lead to DNA double-strand breaks if not properly repaired [52]. The accumulation of such DNA damage results in genomic instability, a hallmark of cancer development [53].

Importantly, recent genomic studies have identified specific mutational signatures in colorectal tumors that are directly attributable to colibactin exposure. These include characteristic base substitutions and insertion–deletion mutations, providing strong molecular evidence linking pks^+^ *E. coli* to human carcinogenesis [54].

Beyond its direct genotoxic effects, pks^+^ *E. coli* also contributes to tumorigenesis through additional mechanisms. Colibactin-producing strains can disrupt the intestinal epithelial barrier, facilitating bacterial translocation and enhancing exposure of the mucosa to microbial antigens. This promotes chronic inflammation, which further amplifies DNA damage through the production of reactive oxygen and nitrogen species [55].

Furthermore, these bacteria influence host cellular processes, including cell cycle regulation and apoptosis. Colibactin-induced DNA damage activates DNA damage response pathways, but persistent exposure may overwhelm repair mechanisms, leading to mutation accumulation and uncontrolled cell proliferation [52,53].

In addition to epithelial effects, pks^+^ *E. coli* contributes to modulation of the tumor microenvironment. Recent evidence suggests that these bacteria can promote a pro-carcinogenic immune landscape by inducing immunosuppressive signaling and altering immune cell function, thereby facilitating tumor progression and potentially contributing to resistance to therapy [55].

Collectively, pks^+^ *E. coli* acts as a direct “initiator” of carcinogenesis through genotoxicity, while also promoting tumor progression via inflammation, barrier disruption, and immune modulation. This multifaceted role distinguishes it from other CRC-associated bacteria and highlights its importance as both a biomarker and a potential therapeutic target in CRC.

*Peptostreptococcus anaerobius* is an emerging bacterial species associated with CRC, increasingly recognized for its ability to promote tumorigenesis through modulation of host signaling pathways and induction of oxidative stress. Studies have shown that *Peptostreptococcus anaerobius* is enriched in CRC tissues compared to normal mucosa, suggesting a functional role in tumor development rather than a passive association [40,56].

A key mechanism by which *Peptostreptococcus anaerobius* contributes to carcinogenesis involves the activation of innate immune signaling pathways, particularly through Toll-like receptors (TLRs). The bacterium interacts with host epithelial cells via surface proteins that engage integrins and activate downstream signaling cascades, including the phosphoinositide 3-kinase (PI3K)–Akt pathway and NF-κB signaling [56]. Activation of these pathways promotes cell proliferation, survival, and inflammatory responses, all of which are hallmarks of tumor progression.

In addition to signaling activation, *Peptostreptococcus anaerobius* induces the production of reactive oxygen species (ROS) within colon epithelial cells. Elevated ROS levels lead to oxidative DNA damage, including base modifications, strand breaks, and genomic instability, which contribute to mutation accumulation and carcinogenesis [40,41].

Moreover, ROS-mediated stress can further activate pro-tumorigenic pathways such as NF-κB and STAT3, creating a feedback loop that sustains inflammation and promotes tumor growth [41]. This oxidative environment also favors epithelial cell transformation and supports tumor progression.

Recent studies have also demonstrated that *Peptostreptococcus anaerobius* can enhance cholesterol biosynthesis in host cells via activation of sterol regulatory element-binding protein 2 (SREBP2), thereby supporting tumor cell proliferation and metabolic reprogramming [56]. This highlights an additional metabolic dimension to its carcinogenic potential.

Collectively, these findings indicate that *Peptostreptococcus anaerobius* contributes to colorectal carcinogenesis through a combination of immune signaling activation, oxidative stress induction, and metabolic modulation.

Importantly, *Peptostreptococcus anaerobius* does not act in isolation but is part of a broader pro-carcinogenic microbial network. Together with bacteria such as *Fusobacterium nucleatum*, enterotoxigenic *Bacteroides fragilis*, and *pks^+^ Escherichia coli*, it contributes to the establishment of a dysbiotic, tumor-promoting microenvironment characterized by chronic inflammation, genomic instability, and immune dysregulation [39]. This synergistic interaction between microbial species and the host plays a critical role in CRC initiation and progression.

**Figure 1:**
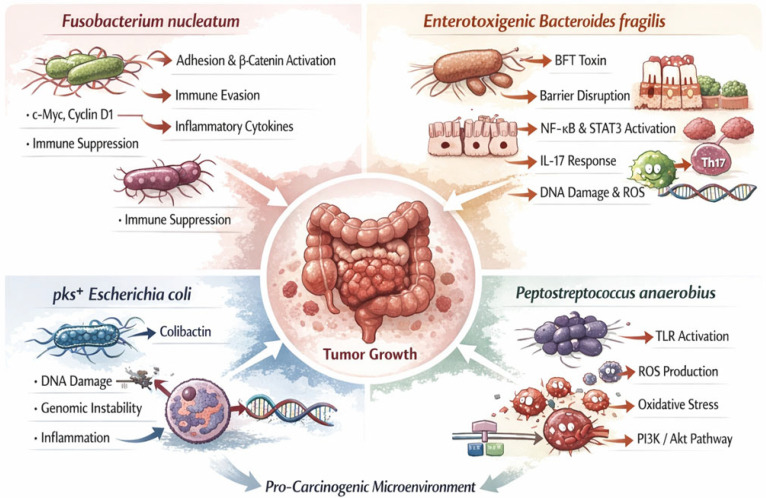
Microbiota-driven mechanisms in colorectal carcinogenesis. *Fusobacterium nucleatum* activates β-catenin signaling and promotes immune evasion. Enterotoxigenic *Bacteroides fragilis* disrupts epithelial integrity and induces NF-κB/STAT3-mediated inflammation. pks^+^ *Escherichia coli* produces colibactin, causing DNA damage and genomic instability. *Peptostreptococcus anaerobius* promotes tumor growth through TLR activation, ROS production, and PI3K/Akt signaling. Together, these bacteria create a pro-carcinogenic microenvironment characterized by inflammation, immune suppression, and genomic instability. This figure represents a conceptual schematic model summarizing the mechanisms discussed in the text and is intended for illustrative purposes only. Figures were created using BioRender.com and adapted by the authors for the purposes of this review.

The role of the gut microbiota in CRC carcinogenesis is presented in Figure 1.

## 3. Mechanisms Linking Microbiota to Colorectal Cancer

The gut microbiota contributes to CRC development through several interconnected biological mechanisms. Among the most important are chronic inflammation, the production of genotoxic metabolites, and modulation of the host immune response. These mechanisms act synergistically, creating a tumor-promoting microenvironment characterized by genomic instability, epithelial dysfunction, and impaired immune surveillance [40,41].

### 3.1. Chronic Inflammation

Chronic inflammation is a central mechanism linking gut microbiota dysbiosis to colorectal carcinogenesis. Under normal conditions, the intestinal immune system maintains tolerance toward commensal bacteria. However, dysbiosis leads to activation of innate immune pathways. Gut microbiota-derived components, including lipopolysaccharides (LPS), flagellin, and peptidoglycans, act as microbe-associated molecular patterns (MAMPs) that are recognized by pattern recognition receptors (PRRs) expressed on intestinal epithelial and immune cells [1,2]. These receptors include Toll-like receptors (TLRs), such as TLR4 (for LPS) and TLR5 (for flagellin), as well as intracellular Nucleotide-binding Oligomerization Domain (NOD), NOD-like receptors (NLRs)- NOD1 and NOD2, which detect peptidoglycan fragments [39,40,57].

Upon activation, these receptors initiate intracellular signaling through adaptor proteins such as Myeloid Differentiation primary response gene 88 protein (MyD88) and TIR-domain-containing adapter inducing interferon-β(TRIF), leading to activation of downstream pathways including NF-κB, Mitogen-Activated Protein Kinase (MAPK), and STAT3. This process represents the first step in translating microbial signals into host inflammatory responses [52].

Importantly, disruption of intestinal barrier integrity facilitates increased exposure of epithelial cells to microbial ligands, amplifying Pattern Recognition Receptor (PRR) activation and promoting carcinogenic signaling [58].

NF-κB is a central transcription factor regulating inflammation, cell survival, and tumor progression. Under basal conditions, NF-κB dimers (p50/p65) are retained in the cytoplasm by inhibitory kappa B proteins (IκB). Microbial stimulation via TLRs activates the IκB kinase (IKK) complex, resulting in phosphorylation and degradation of IκB. This releases NF-κB, allowing its nuclear translocation and transcriptional activation of target genes [59].

NF-κB regulates the expression of numerous pro-inflammatory and pro-tumorigenic mediators, including, cytokines: interleukin-6 (IL-6), tumor necrosis factor alfa (TNF-α), interleukin- 1 (IL-1β), chemokines that recruit immune cells, enzymes such as ciclooxigenase 2 (COX-2) and inducible Nitric Oxide Synthase (iNOS), and anti-apoptotic proteins. Persistent NF-κB activation is a hallmark of CRC and contributes to epithelial proliferation, resistance to apoptosis, angiogenesis, and metastasis [59,60].

Moreover, dysbiosis-associated bacteria can sustain NF-κB activation through continuous stimulation of TLR signaling, reinforcing chronic inflammation and tumor progression [61].

STAT3 is a key transcription factor activated downstream of cytokine signaling, particularly through IL-6 produced during NF-κB activation. IL-6 binds to its receptor and activates Janus kinases (JAKs), which phosphorylate STAT3, enabling its dimerization and nuclear translocation [40]. STAT3 regulates genes involved in cell proliferation (e.g., *cyclin D1*), anti-apoptotic signaling (e.g., B-cell lymphoma 2-*Bcl-2*, B-cell lymphoma-extra large-*Bcl-xL*), angiogenesis (e.g., vascular endothelial growth factor- VEGF), and immune suppression. The NF-κB/IL-6/JAK/STAT3 signaling cascade has been identified as a critical axis in inflammation-driven colorectal carcinogenesis, promoting survival and expansion of tumor-initiating epithelial cells [62]. Persistent STAT3 activation also contributes to immune evasion and tumor microenvironment remodeling.

A defining feature of microbiota-driven CRC is the tight interaction between NF-κB and STAT3 pathways. These pathways form a self-amplifying loop that sustains chronic inflammation and tumorigenesis [40,57].

NF-κB induces IL-6 production, which activates STAT3. Activated STAT3, in turn, enhances the transcription of genes that support inflammation, proliferation, and survival. This creates a positive feedback loop that maintains persistent signaling even in the absence of acute microbial stimuli [62].

Recent studies also highlight the involvement of IL-17–dependent pathways, where microbiota-induced Th17 responses further amplify the NF-κB/STAT3 axis, linking immune activation with epithelial transformation [63].

Chronic activation of NF-κB and STAT3 leads to the establishment of a tumor-promoting microenvironment characterized by continuous cytokine production, recruitment of immune cells (macrophages, neutrophils), and increased production of reactive oxygen and nitrogen species (ROS/RNS). These factors induce DNA damage and genomic instability, promoting tumor initiation and progression [57,60].

Additionally, NF-κB signaling contributes to epithelial–mesenchymal transition (EMT), enhancing tumor invasion and metastasis. It also regulates immune checkpoint expression (e.g., Programmed Death-Ligand 1-PD-L1), facilitating immune evasion [61].

NF-κB activation also disrupts epithelial barrier integrity by altering tight junction proteins, leading to increased intestinal permeability. This allows further translocation of microbial components into the mucosa, reinforcing PRR activation [58].

This establishes a vicious cycle: dysbiosis, PRR activation, NF-κB/STAT3 signaling, inflammation, barrier disruption, and increased microbial exposure. Such feedback loops are central to the persistence of chronic inflammation and CRC progression.

### 3.2. Genotoxic Metabolites in Colorectal Carcinogenesis

Another major mechanism linking gut microbiota to CRC is the production of genotoxic metabolites that directly or indirectly damage host DNA, thereby contributing to genomic instability and tumor initiation. These metabolites include bacterial toxins as well as products of microbial metabolism that interfere with DNA integrity, repair mechanisms, and cellular homeostasis [40,41].

One of the most extensively studied genotoxins is colibactin, produced by pks^+^ *Escherichia coli*. The *pks* genomic island encodes a hybrid polyketide synthase–nonribosomal peptide synthetase system responsible for colibactin biosynthesis [52].

Colibactin exerts its carcinogenic effect by forming covalent DNA adducts and inducing interstrand DNA crosslinks (ICLs). These lesions interfere with DNA replication and transcription, leading to replication fork stalling and the generation of DNA double-strand breaks [52,54].

If DNA repair pathways such as homologous recombination or nucleotide excision repair fail to resolve these lesions, permanent mutations accumulate, including base substitutions and chromosomal rearrangements. Importantly, recent whole-genome sequencing studies have identified a specific mutational signature associated with colibactin exposure in colorectal tumors, providing strong evidence for its direct role in human carcinogenesis [54].

In addition to DNA damage, colibactin can induce cellular senescence and promote a pro-inflammatory microenvironment through the senescence-associated secretory phenotype (SASP), further contributing to tumor progression [52].

*Enterotoxigenic Bacteroides fragilis* (ETBF) produces *Bacteroides fragilis toxin* (BFT), which contributes to carcinogenesis primarily through indirect mechanisms. BFT is a metalloprotease that cleaves E-cadherin, disrupting epithelial cell junctions and increasing intestinal permeability [48].

This barrier disruption allows microbial components to penetrate the mucosa, triggering chronic inflammation. The resulting inflammatory response leads to increased production of ROS and RNS, which can cause oxidative DNA damage, including base modifications, strand breaks, and mutations [48,64].

Additionally, BFT activates signaling pathways such as NF-κB and STAT3, further amplifying inflammation and promoting a tumor-supportive microenvironment. These processes contribute to genomic instability and facilitate tumor initiation and progression [54].

Gut microbiota also contributes to CRC through the transformation of primary bile acids into secondary bile acids, such as deoxycholic acid (DCA) and lithocholic acid (LCA). These metabolites are generated by bacterial enzymes in the colon and have been shown to exert carcinogenic effects [40,65].

Secondary bile acids induce oxidative stress by increasing the production of ROS, leading to DNA damage and lipid peroxidation. They also activate signaling pathways such as NF-κB and Wnt/β-catenin, promoting cell proliferation and inhibiting apoptosis [65].

Moreover, DCA has been shown to disrupt mitochondrial function and induce endoplasmic reticulum stress, further contributing to epithelial injury and carcinogenesis [40].

Hydrogen sulfide (H_2_S), produced by sulfate-reducing bacteria such as *Desulfovibrio* spp., is another important microbial metabolite implicated in CRC. At high concentrations, H_2_S is toxic to epithelial cells and can damage DNA through oxidative mechanisms. H_2_S also inhibits cytochrome c oxidase in mitochondria, impairing cellular respiration and promoting metabolic stress. In addition, it can interfere with DNA repair pathways, further enhancing mutagenesis [40,41].

Other microbial metabolites, including nitrosamines and acetaldehyde, have also been implicated in CRC through their ability to form DNA adducts and induce mutations.

These genotoxic mechanisms are not isolated but interact within the tumor microenvironment. For example, colibactin directly induces DNA damage, inflammation amplifies oxidative stress and mutagenesis, and secondary metabolites further enhance DNA instability. Together, these processes contribute to the accumulation of mutations in key oncogenes and tumor suppressor genes, such as *APC*, *KRAS*, and *TP53*, driving colorectal carcinogenesis. Importantly, recent genomic studies have confirmed that microbial genotoxins leave distinct mutational signatures in CRC genomes, providing strong evidence for a causal link between microbiota and cancer [40,54].

### 3.3. Immune Modulation in Colorectal Carcinogenesis

The gut microbiota plays a fundamental role in shaping and regulating the host immune system, maintaining a dynamic balance between immune tolerance and activation. Under physiological conditions, commensal bacteria contribute to immune homeostasis by promoting regulatory pathways and preventing excessive inflammation. However, in the context of dysbiosis, this balance is disrupted, leading to immune dysregulation that promotes CRC development [40,41].

One of the principal mechanisms by which microbiota contributes to CRC is through suppression of anti-tumor immune responses. Certain bacterial species interfere directly with immune effector cell function, enabling tumor cells to evade immune surveillance. A well-characterized example is *Fusobacterium nucleatum*, which inhibits the cytotoxic activity of natural killer (NK) cells and CD8^+^ T lymphocytes. This effect is mediated by the bacterial Fap2 protein, which binds to the inhibitory T cell Immunoreceptor with Ig and ITIM domains (TIGIT) expressed on immune cells, suppressing their ability to recognize and eliminate tumor cells [3,4]. In addition, *Fusobacterium nucleatum* can impair T cell activation and reduce interferon-γ production, further weakening anti-tumor immunity [17].

At the same time, other bacteria promote pro-inflammatory immune responses that paradoxically support tumor growth. *Enterotoxigenic Bacteroides fragilis* (ETBF) induces a Th17-mediated immune response characterized by increased production of IL-17. This cytokine plays a critical role in tumor-promoting inflammation by enhancing immune cell recruitment, activating NF-κB and STAT3 signaling pathways, and stimulating angiogenesis and epithelial cell proliferation [66].

Chronic activation of Th17 responses contributes to a persistent inflammatory microenvironment that favors tumor progression. Dysbiosis also leads to the expansion of immunosuppressive cell populations within the tumor microenvironment. Tregs and myeloid-derived suppressor cells (MDSCs) are particularly important in this context. These cells suppress effector T cell responses through the production of anti-inflammatory cytokines such as IL-10 and transforming growth factor-β (TGF-β), thereby inhibiting effective anti-tumor immunity and promoting tumor immune evasion [40].

Furthermore, the gut microbiota influences antigen presentation and cytokine signaling networks. Dysbiosis can impair dendritic cell function, reducing their ability to present tumor antigens and activate T cells. In parallel, microbial signals modulate cytokine production, shifting the balance toward pro-tumorigenic pathways, such as increased IL-6/STAT3 signaling and reduced interferon-mediated immune responses [40,41].

The microbiota also plays a critical role in shaping the tumor microenvironment by regulating immune cell recruitment and activation. This results in a tumor-permissive environment characterized by chronic inflammation, immune suppression, and reduced immune surveillance. Importantly, emerging evidence indicates that gut microbiota composition significantly influences the efficacy of cancer immunotherapies, particularly immune checkpoint inhibitors. Specific microbial profiles have been associated with improved therapeutic responses, while dysbiosis may contribute to resistance by promoting immunosuppressive pathways. This highlights the potential of microbiota-targeted interventions as adjunct strategies in personalized cancer therapy [40,67].

Table 1 presents the effects of individual bacterial species and the broader impact of dysbiotic microbiota. While specific bacteria exert distinct mechanisms, colorectal carcinogenesis is driven by a synergistic microbial community in which multiple species collectively promote inflammation, immune suppression, and tumor progression.

## 4. Probiotics, Prebiotics and Synbiotics

Probiotics are defined as live microorganisms that confer a health benefit to the host when administered in adequate amounts [69]. Common probiotic genera include *Lactobacillus*, *Bifidobacterium*, and *Saccharomyces*. Their mechanisms of action include competitive exclusion of pathogenic bacteria, production of antimicrobial substances such as bacteriocins and lactic acid, modulation of immune responses through the stimulation of anti-inflammatory cytokines, and enhancement of intestinal barrier function. However, a key limitation of probiotics is their variable colonization efficiency, as many strains do not persist long-term in the gut due to competition with resident microbiota [70,71].

Prebiotics are non-digestible food components that selectively stimulate the growth and/or activity of beneficial microorganisms in the gut [72]. Typical examples include inulin, fructooligosaccharides (FOS), and galactooligosaccharides (GOS). Their primary mechanisms include selective fermentation by beneficial bacteria; increased production of SCFAs, especially butyrate; improvement of epithelial barrier integrity; and indirect modulation of immune responses [70,73]. Unlike probiotics, prebiotics do not introduce new microorganisms but rather enhance the activity of the existing microbiota [72].

Synbiotics are defined as combinations of probiotics and prebiotics that are designed to work together to improve gut microbial balance and host health [1]. Their key advantages include improved survival and colonization of probiotic strains, enhanced metabolic activity of beneficial bacteria, increased production of SCFAs, and stronger modulation of immune and inflammatory pathways. Synbiotics are increasingly considered superior in certain contexts because they address both microbial composition and function simultaneously, which is essential in conditions involving dysbiosis and chronic inflammation [21,40,70,74].

According to the International Scientific Association for Probiotics and Prebiotics (ISAPP), synbiotics can be classified into two main types: complementary synbiotics, in which probiotics and prebiotics act independently, and synergistic synbiotics, where the prebiotic component is specifically selected to enhance the growth and activity of the administered probiotic strain [21].

This classification is critical for understanding their biological effects, clinical applications, and therapeutic potential, particularly in microbiota-related diseases such as colorectal cancer [70,74,75].

Complementary synbiotics consist of probiotic and prebiotic components that act independently, each contributing to host health through distinct mechanisms. In these formulations, the probiotic exerts beneficial effects through mechanisms such as competitive exclusion of pathogens, production of antimicrobial compounds, and modulation of immune responses. Meanwhile, the prebiotic selectively stimulates the growth of beneficial endogenous microbiota, enhancing microbial diversity and metabolic activity [21,70,76].

Importantly, the prebiotic component in complementary synbiotics is not specifically designed to be utilized by the administered probiotic strain, but rather targets the broader microbial ecosystem. However, a key limitation of probiotics is their variable colonization efficiency, as many strains do not persist long-term in the gut due to competition with resident microbiota [21,75]. As a result, the effects of complementary synbiotics are generally additive, reflecting the independent contributions of each component rather than a direct interaction between them.

Recent studies highlight that complementary synbiotics can improve gut microbiota composition by increasing beneficial genera such as *Bifidobacterium* and *Lactobacillus*, while reducing pathogenic bacteria. They also contribute to increased production of SCFAs, which are essential for maintaining intestinal barrier integrity and modulating immune responses [71,73,77].

However, one limitation of complementary synbiotics is their lack of specificity, as the prebiotic substrate may be utilized by multiple microbial species, not exclusively the administered probiotic. This may reduce the efficiency of targeted microbiota modulation and lead to variable clinical outcomes [75].

In contrast, synergistic synbiotics are designed to establish a direct metabolic interaction between the probiotic and prebiotic components. In these formulations, the prebiotic substrate is selectively utilized by the co-administered probiotic strain, thereby enhancing its survival, colonization, and functional activity in the gastrointestinal tract [21,75].

This targeted relationship leads to a true synergistic effect, where the combined action of the probiotic and prebiotic exceeds their individual effects. The prebiotic acts as a specific energy source for the probiotic, improving its persistence and metabolic output, including increased production of beneficial metabolites such as SCFAs [73,78].

At the molecular level, synergistic synbiotics exert their beneficial effects by modulating several key pathways involved in intestinal homeostasis and inflammation. One of the primary mechanisms is the enhancement of SCFA production, particularly butyrate, which serves as a major energy source for colonocytes and contributes to the maintenance of epithelial barrier integrity. This leads to improved mucosal protection and reduced intestinal permeability [79,80].

In addition, synergistic synbiotics influence epigenetic regulation through the inhibition of histone deacetylases (HDACs). This mechanism results in the suppression of pro-inflammatory gene expression and promotes anti-tumor activity by inducing apoptosis and inhibiting uncontrolled cell proliferation [81,82].

Furthermore, these formulations downregulate major pro-inflammatory signaling pathways, including NF-κB and STAT3, which are critically involved in chronic inflammation and colorectal carcinogenesis. Inhibition of these pathways leads to a decrease in the production of inflammatory cytokines and limits the establishment of a tumor-promoting microenvironment [83,84].

Finally, synergistic synbiotics modulate immune responses by promoting Treg activity while simultaneously reducing Th17-mediated inflammatory responses. This shift toward an anti-inflammatory immune profile contributes to immune homeostasis and may play a protective role against tumor development [85,86].

Recent evidence suggests that synergistic synbiotics may provide more consistent and targeted microbiota modulation compared to complementary formulations. They can also overcome limitations associated with probiotic colonization, as the prebiotic substrate enhances engraftment and persistence of the administered strains [78,87,88].

Furthermore, studies indicate that synergistic synbiotics may be particularly effective in disease contexts such as CRC, where targeted modulation of microbial metabolism and immune signaling pathways is required [74,87,88,89].

The distinction between complementary and synergistic synbiotics has important implications for their clinical application. Recent research suggests that both types can modulate immune responses, increase SCFA production, and reduce inflammatory cytokines such as TNF-α and IL-6. However, synergistic synbiotics may demonstrate superior efficacy in specific conditions due to their targeted design [77,87,90]. Interestingly, some studies indicate that distinguishing between the two types can be challenging in vivo, as prebiotic substrates may still be utilized by endogenous microbiota, even in synergistic formulations. This highlights the complexity of microbiome interactions and the need for further research [75].

### Synbiotic Combinations

Commonly used synbiotic formulations consist of specific probiotic strains combined with prebiotic substrates that support their growth and metabolic activity in the gastrointestinal tract. One of the most widely studied combinations includes *Bifidobacterium* spp. (e.g., *Bifidobacterium longum*, *Bifidobacterium breve*) together with fructooligosaccharides (FOS) or inulin. These prebiotics selectively stimulate *Bifidobacterium* growth, leading to increased production of SCFAs, particularly butyrate, which contributes to epithelial barrier integrity and anti-inflammatory effects [74,76].

Another frequently used combination involves *Lactobacillus* species, such as *Lactobacillus rhamnosus* or *Lactobacillus acidophilus*, paired with galactooligosaccharides (GOS). This formulation enhances probiotic survival and promotes immune modulation, including the reduction in pro-inflammatory cytokines and improvement of mucosal immunity. Multi-strain synbiotic formulations combining *Lactobacillus*, *Bifidobacterium*, and *Streptococcus thermophilus* with prebiotic fibers such as inulin or resistant starch are also widely used. These combinations aim to increase microbial diversity and functional resilience of the gut microbiota, contributing to improved metabolic activity and reduced dysbiosis [76,78]. In clinical settings, combinations such as *Bifidobacterium lactis* with resistant starch or inulin have demonstrated the ability to enhance butyrate production and reduce inflammation, which is particularly relevant in CRC prevention and management [73,74]. More recent studies highlight that synbiotics can reduce postoperative infections, improve chemotherapy tolerance, and modulate immune responses in CRC patients, further supporting their therapeutic potential [73,78,90].

**Figure 2:**
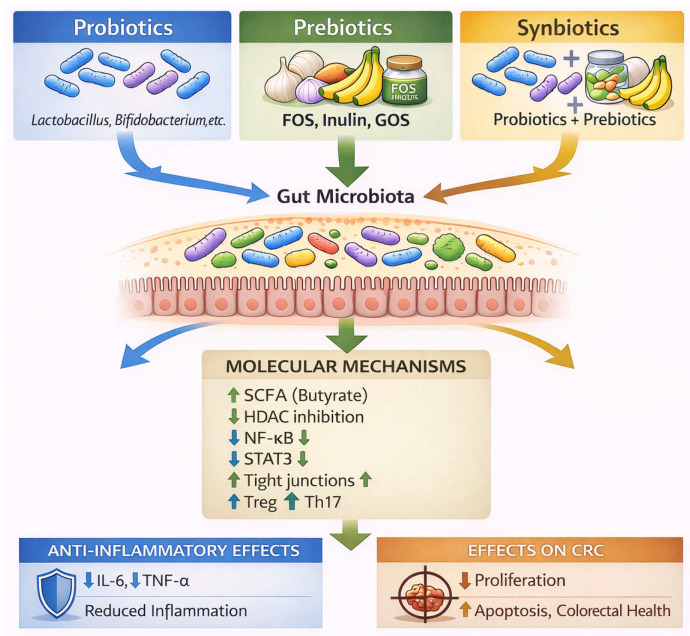
Modulation of Gut Microbiota and Molecular Mechanisms of Synbiotics in CRC. Probiotics (e.g., *Lactobacillus*, *Bifidobacterium*) and prebiotics (e.g., FOS, inulin, GOS) act individually or synergistically as synbiotics to restore microbial balance. This leads to increased production of SCFAs, particularly butyrate, resulting in histone deacetylase (HDAC) inhibition, suppression of inflammatory pathways (NF-κB, STAT3), and enhancement of intestinal barrier integrity through tight junctions. These molecular changes promote immune regulation (Treg/Th17 balance), reduce pro-inflammatory cytokines (IL-6, TNF-α), and ultimately decrease tumor cell proliferation while increasing apoptosis, contributing to improved colorectal health. This figure represents a conceptual schematic model summarizing the mechanisms discussed in the text and is intended for illustrative purposes only. Figures were created using BioRender.com and adapted by the authors for the purposes of this review.

Figure 2 illustrates the role of probiotics, prebiotics, and synbiotics in modulating gut microbiota and their downstream effects in CRC.

## 5. Mechanisms of Action of Synbiotics in Colorectal Cancer: Integration with Molecular Signaling Pathways

CRC is a multifactorial disease arising from dynamic interactions between gut microbiota, inflammatory processes, metabolic reprogramming, and dysregulated host signaling pathways. Synbiotics exert protective effects through modulation of gut microbiota, production of bioactive metabolites, regulation of immune responses, and maintenance of intestinal barrier integrity [16,91].

While these mechanisms describe the functional impact of synbiotics, their biological effects are ultimately mediated through intracellular signaling pathways. In particular, the Wnt/β-catenin, NF-κB, and phosphoinositide 3-kinase/mammalian Target of Rapamycin (PI3K/Akt/mTOR) pathways represent central nodes linking microbiota-derived signals to tumor cell behavior. Therefore, understanding how synbiotics influence these pathways provides a mechanistic bridge between microbiota modulation and cancer inhibition.

To improve conceptual clarity, the mechanisms discussed in this section can be broadly categorized into primary and secondary effects. The primary mechanisms involve modulation of gut microbiota composition and enhanced production of SCFAs, particularly butyrate, which represent the central mediators of synbiotic activity in CRC. These upstream events subsequently influence multiple interconnected downstream processes, including suppression of NF-κB-mediated inflammation, reduction in oxidative stress, improvement of epithelial barrier integrity, and regulation of apoptosis and proliferation through Wnt/β-catenin and PI3K/Akt/mTOR signaling pathways. Thus, rather than acting as isolated mechanisms, these processes form an integrated biological network through which synbiotics exert anti-tumor effects.

### 5.1. Functional Mechanisms of Synbiotics

Synbiotics act at multiple levels within the intestinal microenvironment.

#### 5.1.1. Modulation of Gut Microbiota

One of the most important and well-characterized mechanisms by which synbiotics exert their protective effects in CRC is through the modulation of gut microbiota composition. The intestinal microbiome represents a highly dynamic and complex ecosystem composed of trillions of microorganisms, including bacteria, archaea, viruses, and fungi, which coexist in a finely regulated equilibrium known as eubiosis. This equilibrium is essential for maintaining intestinal homeostasis, metabolic balance, and immune function [40,92].

Disruption of this balance, referred to as dysbiosis, has been strongly associated with colorectal carcinogenesis. Dysbiosis is characterized by decreased microbial diversity, depletion of beneficial commensal bacteria such as *Faecalibacterium prausnitzii*, *Bifidobacterium*, and *Lactobacillus*, and enrichment of pathogenic or pro-carcinogenic species, including *Fusobacterium nucleatum*, *Enterotoxigenic Bacteroides fragilis*, and *pks+ Escherichia coli* [40]. These alterations contribute to tumor development through increased inflammation, production of genotoxic metabolites, and impairment of epithelial barrier function [16,22,93].

Synbiotics are specifically designed to restore microbial homeostasis by simultaneously introducing beneficial microorganisms and providing selective substrates that enhance their survival, colonization, and metabolic activity. This dual approach allows for a more efficient and sustained modulation of gut microbiota compared to probiotics or prebiotics alone, as it supports both microbial engraftment and ecological stability [21].

Synbiotic supplementation promotes the proliferation and metabolic activity of beneficial bacterial genera such as *Lactobacillus* and *Bifidobacterium*, which are key regulators of intestinal homeostasis. These microorganisms contribute to gut health through multiple mechanisms, including competitive exclusion of pathogens, production of antimicrobial compounds (e.g., bacteriocins, lactic acid), and modulation of host immune responses [21,71].

Prebiotic substrates such as FOS, inulin, and GOS are selectively fermented by these beneficial bacteria, leading to their expansion and increased metabolic output. This selective enrichment enhances microbial diversity and resilience, allowing the microbiota to better resist colonization by pathogenic species and maintain functional stability [21,70,71]. Increased microbial diversity is particularly relevant in CRC, as numerous studies have demonstrated that reduced diversity is a hallmark of tumor-associated dysbiosis and is associated with disease progression and poor prognosis [40]. In addition to promoting beneficial microbes, synbiotics play a crucial role in suppressing pathogenic and pro-carcinogenic bacteria. Species such as *Fusobacterium nucleatum*, *Escherichia coli* (pks^+^ strains), and *Bacteroides fragilis* are known to produce virulence factors, adhesins, and genotoxins that directly contribute to tumor initiation and progression [22,54,93].

Synbiotics inhibit these harmful microorganisms through several complementary mechanisms, including competitive exclusion, where beneficial bacteria occupy ecological niches and limit pathogen adhesion; reduction in luminal pH through SCFA production; and production of antimicrobial substances, including bacteriocins and organic acids. These mechanisms reduce the abundance of tumor-promoting bacteria and limit their ability to activate oncogenic pathways such as NF-κB, STAT3, and Wnt/β-catenin signaling [16,54,73].

Beyond compositional changes, synbiotics also induce profound functional modifications in the gut microbiome. CRC-associated dysbiosis is not only defined by altered bacterial taxa but also by disrupted metabolic activity, including increased production of carcinogenic metabolites such as secondary bile acids, hydrogen sulfide, and ROS [54,73].

Synbiotics promote beneficial metabolic pathways, particularly those involved in carbohydrate fermentation and SCFA production, while suppressing pathways associated with toxin generation and carcinogen formation. This metabolic reprogramming leads to increased production of protective metabolites (e.g., butyrate), reduced synthesis of genotoxic compounds, and decreased oxidative stress. Such functional restoration is essential for re-establishing a protective intestinal environment and preventing tumor-promoting processes [22,73,94].

The gut microbiota plays a fundamental role in regulating host–microbiota crosstalk, which involves continuous communication between microbial communities and host cells through metabolites, microbial-associated molecular patterns (MAMPs), and signaling molecules. Synbiotics modulate this bidirectional interaction by shaping microbial communities that favor anti-inflammatory and immunoregulatory responses. A balanced microbiota promotes immune tolerance and controlled immune activation, whereas dysbiosis leads to persistent stimulation of pattern recognition receptors (e.g., TLRs), resulting in chronic inflammation and tumor-promoting signaling [40,54,95].

Through microbiota modulation, synbiotics influence key host processes, including immune cell differentiation and cytokine production, epithelial cell proliferation and apoptosis, maintenance of barrier integrity, and regulation of inflammatory signaling pathways. These effects collectively contribute to restoring immune homeostasis and reducing tumor-promoting inflammation [54,71,95].

Another critical mechanism of synbiotics involves reducing microbial virulence and carcinogenic potential. Pathogenic bacteria associated with CRC express a wide range of virulence factors, including toxins, adhesins, and enzymes that promote DNA damage, inflammation, and epithelial disruption [16,93]. Synbiotics can downregulate these virulence factors by altering microbial composition and metabolic activity. For instance, probiotic strains have been shown to reduce the activity of bacterial enzymes such as β-glucuronidase, nitroreductase, and azoreductase, which are involved in the conversion of procarcinogens into active carcinogens [70]. Additionally, synbiotics may interfere with bacterial adhesion and biofilm formation, thereby reducing microbial persistence and pathogenicity. This contributes to lowering the overall carcinogenic potential of the gut microbiota and limiting tumor progression.

Collectively, these microbiota-related changes represent the primary upstream mechanism through which synbiotics influence CRC progression, subsequently affecting inflammatory, oxidative, metabolic, and proliferative signaling pathways.

Table 2 presents the roles of synbiotics in gut homeostasis.

#### 5.1.2. Production of Beneficial Metabolites

One of the most important mechanisms through which synbiotics exert anti-carcinogenic effects in CRC is the enhanced production of beneficial microbial metabolites, particularly SCFAs, including acetate, propionate, and butyrate. These metabolites are generated through the fermentation of non-digestible carbohydrates (prebiotics such as inulin, fructooligosaccharides and dietary fiber) by commensal gut bacteria, mainly *Lactobacillus*, *Bifidobacterium*, *Faecalibacterium prausnitzii*, and *Roseburia* spp. This metabolic process represents a key link between diet, microbiota composition, and host cellular signaling in CRC pathogenesis [97,98,99].

Butyrate, in particular, plays a pivotal role as both an energy substrate for colonocytes and a potent signaling molecule. Under physiological conditions, butyrate is the primary energy source for healthy colon epithelial cells, supporting mitochondrial β-oxidation and maintaining epithelial homeostasis. However, in CRC cells, which preferentially rely on glycolysis (the “Warburg effect”), butyrate accumulates intracellularly instead of being oxidized. This accumulation leads to its function as a histone deacetylase (HDAC) inhibitor, inducing profound epigenetic changes that regulate gene [100,101].

Butyrate exerts potent anti-inflammatory effects. Butyrate plays a central role in NF-κB inhibition through both direct and indirect mechanisms. One of its key mechanisms involves the inhibition of histone deacetylases, which alters chromatin structure and suppresses transcription of NF-κB target genes, leading to epigenetic modulation of gene expression. Through HDAC inhibition, butyrate promotes hyperacetylation of histone proteins, resulting in the activation of tumor suppressor genes (e.g., *p21*, *p27*) and repression of oncogenes [102,103,104,105].

Through this pathway, butyrate suppresses the transcription of pro-inflammatory cytokines while promoting the differentiation and expansion of Tregs, which are essential for immune tolerance and control of excessive inflammatory responses [43].

Additionally, SCFAs play a crucial role in immune modulation. Butyrate enhances the differentiation and expansion of Tregs through epigenetic regulation of the *FOXP3* gene, leading to suppression of excessive inflammatory responses. This is particularly important in CRC, where chronic inflammation drives tumor progression. By increasing Treg populations and reducing Th17-mediated inflammation, SCFAs help restore immune homeostasis within the tumor microenvironment [106].

Another important mechanism involves the regulation of intestinal barrier integrity. Butyrate stimulates the expression of tight junction proteins such as occludin and claudins, thereby strengthening the epithelial barrier and preventing translocation of bacterial components such as LPS. Reduced LPS translocation leads to decreased activation of TLR4-mediated inflammatory signaling pathways, which are known to promote NF-κB activation and tumor progression [107,108,109].

Moreover, butyrate exerts direct pro-apoptotic effects in CRC cells by activating intrinsic apoptotic pathways. It increases mitochondrial membrane permeability, promotes cytochrome c release, and activates caspases (caspase-3 and caspase-9), leading to programmed cell death. At the same time, it downregulates anti-apoptotic proteins such as Bcl-2 and Bcl-xL, which are often overexpressed in CRC [110].

SCFAs also influence oxidative stress and genomic stability. Butyrate enhances antioxidant defense systems by increasing the activity of enzymes such as superoxide dismutase (SOD) and catalase, while reducing the production of ROS. This protects epithelial cells from DNA damage and reduces mutation rates associated with carcinogenesis [111,112].

At the molecular level, butyrate interacts with several G-protein-coupled receptors (GPCRs), including GPR41, GPR43, and GPR109A, expressed on epithelial and immune cells. Activation of these receptors leads to anti-inflammatory signaling cascades, including the suppression of NF-κB nuclear translocation and decreased production of pro-inflammatory cytokines. In particular, GPR109A activation induces anti-inflammatory responses and promotes epithelial cell differentiation, thereby contributing to tumor suppression [113].

Importantly, synbiotics amplify all these beneficial effects by simultaneously providing substrates (prebiotics) and functional bacteria (probiotics) that optimize SCFA production. This synergistic interaction leads to sustained butyrate production, improved microbial balance, and long-term modulation of tumor-related pathways.

In summary, SCFAs—especially butyrate—represent a central mechanistic link between synbiotics and CRC prevention. Through a combination of metabolic, epigenetic, immunological, and anti-inflammatory actions, butyrate effectively suppresses tumor initiation and progression, making it a key target in microbiome-based therapeutic strategies.

Moreover, SCFAs act as central mediators linking microbiota modulation to downstream regulation of inflammation, oxidative stress, apoptosis, and oncogenic signaling in CRC.

#### 5.1.3. Anti-Inflammatory Effects

Chronic inflammation is a central driver of CRC initiation and progression, with the transcription factor NF-κB playing a pivotal role in orchestrating tumor-promoting inflammatory responses. Under physiological conditions, NF-κB regulates immune defense and cellular homeostasis; however, its persistent activation in CRC leads to uncontrolled expression of pro-inflammatory cytokines, anti-apoptotic proteins, and genes involved in proliferation, angiogenesis, and metastasis [114]. Synbiotics exert potent anti-inflammatory effects by targeting multiple levels of NF-κB signaling, thereby disrupting the inflammatory–tumorigenic feedback loop [115,116].

At the molecular level, NF-κB activation is typically initiated through pattern recognition receptors such as Toll-like receptor 4, which are stimulated by microbial components like lipopolysaccharide (LPS). This interaction triggers the MyD88-dependent signaling cascade, leading to activation of the IκB kinase complex. IKK phosphorylates the inhibitor protein IκBα, marking it for ubiquitination and proteasomal degradation. As a result, NF-κB (most commonly the p65/p50 heterodimer) translocates into the nucleus, where it induces transcription of pro-inflammatory genes including IL-6, TNF-α, COX-2, and iNOS [54,117,118,119,120]. In CRC, this pathway is often constitutively active, sustaining a chronic inflammatory microenvironment that promotes tumor growth.

Butyrate plays a central role in NF-κB inhibition through both direct and indirect mechanisms. One of its key actions is the inhibition of histone deacetylases (HDACs), which alters chromatin structure and suppresses transcription of NF-κB target genes. Moreover, butyrate stabilizes IκBα, preventing its degradation and thereby retaining NF-κB in the cytoplasm. This effectively blocks NF-κB nuclear translocation and downstream gene expression. In parallel, butyrate reduces the phosphorylation of the p65 subunit, further limiting its transcriptional activity [121,122,123,124].

Synbiotics counteract tumor growth through several complementary mechanisms. First, by modulating gut microbiota composition, they reduce the abundance of Gram-negative bacteria responsible for LPS production, thereby decreasing TLR4 activation. This upstream effect leads to reduced stimulation of the NF-κB pathway [52]. Additionally, synbiotics increase beneficial bacteria that produce anti-inflammatory metabolites, particularly SCFAs, such as butyrate.

Synbiotics also modulate the immune system, shifting the balance from a pro-inflammatory to an anti-inflammatory state. They promote the expansion of Tregs and increase the production of anti-inflammatory cytokines such as IL-10, while reducing pro-inflammatory mediators like IL-6 and TNF-α. This immune modulation disrupts the positive feedback loop between NF-κB activation and cytokine signaling, particularly the NF-κB/IL-6/STAT3 axis, which is known to sustain tumor-promoting inflammation in CRC [106].

Furthermore, synbiotics enhance intestinal barrier integrity, reducing epithelial permeability and preventing translocation of microbial products such as LPS into the systemic circulation. This decreases activation of TLR-mediated signaling pathways and further attenuates NF-κB activation [54]. By maintaining barrier function, synbiotics limit one of the key triggers of chronic inflammation in CRC.

Importantly, inhibition of NF-κB signaling has downstream effects on multiple cancer-related processes. Reduced NF-κB activity leads to decreased expression of genes involved in cell proliferation (Cyclin D1, c-Myc), survival (Bcl-2, Bcl-xL), angiogenesis (VEGF), and invasion and metastasis (MMPs) [125,126].

Thus, the anti-inflammatory effects of synbiotics extend beyond inflammation control to directly influence tumor biology.

Importantly, suppression of NF-κB signaling not only reduces inflammation but also indirectly influences oxidative stress, epithelial integrity, and proliferative pathways, highlighting the interconnected nature of synbiotic-mediated effects in CRC.

**Figure 3:**
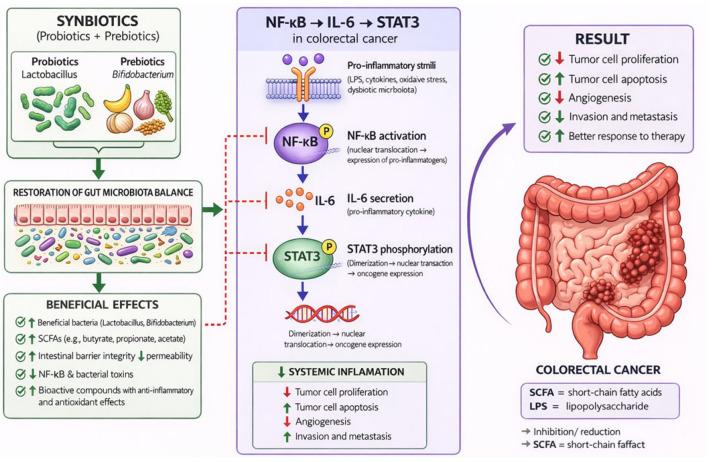
Synbiotic modulation of the NF-κB/IL-6/STAT3 axis in CRC. Synbiotics restore gut microbiota balance, increasing beneficial bacteria and the production of SCFAs, while reducing pathogenic bacteria and endotoxin levels. These changes inhibit key pro-inflammatory pathways, particularly the NF-κB/IL-6/STAT3 signaling axis. Synbiotics suppress NF-κB activation, decrease IL-6 secretion, and limit STAT3 phosphorylation and nuclear translocation, thereby reducing the expression of oncogenic genes involved in cell proliferation, survival, angiogenesis, and metastasis. Overall, these effects lead to decreased systemic inflammation, reduced tumor progression, increased apoptosis, and improved response to therapy in CRC. This figure represents a conceptual schematic model summarizing the mechanisms discussed in the text and is intended for illustrative purposes only. Figures were created using BioRender.com and adapted by the authors for the purposes of this review.

Figure 3 illustrates the mechanisms by which synbiotics influence inflammatory signaling in CRC.

#### 5.1.4. Regulation of Apoptosis and Proliferation

A defining hallmark of CRC is the imbalance between uncontrolled tumor cell proliferation and impaired apoptosis. Synbiotics exert significant anti-tumor effects by restoring this balance through coordinated regulation of apoptotic pathways, cell cycle checkpoints, and oncogenic signaling cascades. These effects are mediated both directly—through microbial metabolites such as SCFAs, particularly butyrate—and indirectly, via modulation of inflammation and key transcription factors such as NF-κB and STAT3 [127,128].

At the cellular level, apoptosis can be induced via two major pathways: the intrinsic (mitochondrial) and extrinsic (death receptor-mediated) pathways. Synbiotics primarily activate the intrinsic pathway through the action of butyrate, which accumulates in CRC cells due to altered tumor metabolism (Warburg effect) [101]. This accumulation leads to inhibition of HDACs, resulting in epigenetic reprogramming and activation of pro-apoptotic genes [123].

Butyrate induces mitochondrial dysfunction by altering the balance between pro- and anti-apoptotic members of the Bcl-2 protein family. Specifically, it upregulates pro-apoptotic proteins such as Bax and Bak and it downregulates anti-apoptotic proteins such as Bcl-2 and Bcl-xL. This shift leads to increased mitochondrial outer membrane permeability (MOMP), resulting in the release of cytochrome c into the cytoplasm. Cytochrome c then forms a complex with Apaf-1 and procaspase-9 (apoptosome), triggering activation of caspase-9, followed by downstream executioner caspases such as caspase-3 and caspase-7. This cascade ultimately leads to apoptotic cell death [129,130].

In parallel, butyrate increases the expression of tumor suppressor genes such as *p53* and *p21*, which further promote apoptosis and inhibit proliferation [123].

Synbiotics may also enhance the extrinsic apoptotic pathway by increasing the expression of death receptors such as Fas (CD95) and TNF-Related Apoptosis-Inducing Ligand (TRAIL) receptors (DR4/DR5) on tumor cells. Activation of these receptors leads to recruitment of adaptor proteins and activation of caspase-8, which can directly activate executioner caspases or amplify the intrinsic pathway through Bid cleavage [82].

A central mechanism underlying these effects is HDAC inhibition by butyrate. By increasing histone acetylation, butyrate promotes transcription of genes involved in apoptosis while suppressing genes that drive proliferation. This includes activation of cyclin-dependent kinase inhibitors (p21, p27) and repression of oncogenes such as *c-Myc*. This epigenetic modulation leads to both apoptosis induction and cell cycle arrest [123].

Synbiotics inhibit proliferation by targeting key signaling pathways that regulate cell growth and survival. In the meantime, chronic activation of NF-κB promotes proliferation and inhibits apoptosis. Synbiotics reduce NF-κB activity by decreasing LPS-induced TLR4 signaling and inhibiting nuclear translocation of NF-κB. This results in reduced expression of proliferation-associated genes such as Cyclin D1 and c-Myc [127].

Synbiotics also affect tumor metabolism. By increasing butyrate levels, they counteract the glycolytic phenotype of cancer cells. The accumulation of butyrate disrupts metabolic homeostasis and induces metabolic stress, which further contributes to growth inhibition and apoptosis [101].

The combined effects of synbiotics on apoptosis and proliferation result in increased tumor cell death, reduced tumor growth, and decreased resistance to therapy. Importantly, these mechanisms are interconnected. For example, inhibition of NF-κB enhances apoptosis, HDAC inhibition affects both apoptosis and proliferation, and microbial metabolites regulate multiple oncogenic pathways simultaneously. Thus, synbiotics act as multi-target modulators of cancer cell fate [80,128].

These effects on apoptosis and proliferation largely reflect downstream consequences of microbiota-derived metabolites and inflammatory pathway modulation, rather than isolated mechanisms.

#### 5.1.5. Reduction in Oxidative Stress

CRC is also influenced by genetic variability, including polymorphisms in genes involved in DNA repair and folate metabolism, which can modulate the cellular response to oxidative stress. These genetic alterations may impair DNA repair capacity and increase susceptibility to oxidative DNA damage, thereby contributing to genomic instability and promoting colorectal carcinogenesis [131,132,133]. Oxidative stress represents a critical contributor to colorectal carcinogenesis, arising from an imbalance between the production of ROS and the capacity of antioxidant defense systems. In CRC, excessive ROS levels promote genomic instability, DNA damage, and activation of oncogenic signaling pathways. Synbiotics exert protective effects by reducing oxidative stress and limiting DNA damage through multiple interconnected mechanisms involving microbiota modulation, antioxidant activity, and regulation of inflammatory pathways [91,134].

At the cellular level, ROS, including superoxide anion (O_2_^−^), hydrogen peroxide (H_2_O_2_), and hydroxyl radicals (^•^OH), are generated through mitochondrial respiration, inflammatory cell activity, and microbial metabolism. Under physiological conditions, ROS act as signaling molecules; however, in CRC, chronic inflammation and dysbiosis lead to excessive ROS production. This results in oxidative damage to DNA, including base modifications (e.g., 8-oxo-deoxyguanosine formation), single- and double-strand breaks, and chromosomal instability [135].

A key source of oxidative stress in CRC is the gut microbiota. Certain pathogenic bacteria, such as *Escherichia coli* strains producing colibactin and enterotoxigenic *Bacteroides fragilis*, can directly induce DNA damage and promote tumorigenesis. These bacteria activate inflammatory pathways, including NF-κB, leading to increased production of ROS and RNS by immune cells [91,136]. This establishes a vicious cycle in which inflammation and oxidative stress mutually reinforce each other.

Synbiotics counteract these processes primarily by restoring microbial balance. By increasing beneficial bacteria such as *Lactobacillus* and *Bifidobacterium*, synbiotics reduce the abundance of ROS-producing pathogenic species and decrease the generation of genotoxic metabolites. This shift in microbial composition leads to a reduction in oxidative stress at its source [16].

Another central mechanism involves the production of SCFAs, particularly butyrate. Butyrate exerts antioxidant effects by enhancing the expression and activity of endogenous antioxidant enzymes, including SOD, catalase, and glutathione peroxidase. These enzymes neutralize ROS and prevent oxidative damage to cellular macromolecules. Additionally, butyrate activates the Nrf2 (nuclear factor erythroid 2–related factor 2) pathway, a master regulator of antioxidant responses. Upon activation, Nrf2 translocates to the nucleus and induces the expression of genes encoding antioxidant and detoxifying enzymes, thereby strengthening cellular defense mechanisms against oxidative stress [113].

Furthermore, synbiotics indirectly reduce oxidative stress by suppressing chronic inflammation. Since inflammatory signaling pathways such as NF-κB stimulate ROS production through activation of enzymes like NADPH oxidase and iNOS, inhibition of these pathways results in decreased ROS generation. By downregulating NF-κB signaling, synbiotics reduce both cytokine production and oxidative stress, breaking the link between inflammation and carcinogenesis [134,137].

Synbiotics also contribute to maintaining intestinal barrier integrity, which plays a crucial role in preventing oxidative damage. A compromised intestinal barrier allows translocation of microbial products such as lipopolysaccharide, which activates immune cells and triggers ROS production. By strengthening tight junctions and enhancing mucosal defense, synbiotics limit LPS translocation and reduce systemic oxidative stress [137].

At the genomic level, synbiotics help preserve DNA integrity by reducing oxidative DNA damage and improving DNA repair mechanisms. Reduced ROS levels decrease the formation of mutagenic lesions such as 8-oxo-dG, which is associated with G→T transversions in oncogenes and tumor suppressor genes. Additionally, SCFAs have been shown to modulate the expression of genes involved in DNA repair pathways, further protecting against genomic instability [113,128].

Another important aspect is the reduction in lipid peroxidation, a process in which ROS attack cell membrane lipids, generating toxic by-products such as malondialdehyde (MDA). These products can form DNA adducts and contribute to mutagenesis. Synbiotics decrease lipid peroxidation by reducing ROS levels and enhancing antioxidant defenses, thereby preventing secondary DNA damage [135].

Importantly, oxidative stress is closely linked to tumor progression and resistance to therapy. High ROS levels can activate signaling pathways that promote proliferation, angiogenesis, and survival. By reducing oxidative stress, synbiotics not only prevent tumor initiation but also enhance the sensitivity of cancer cells to therapeutic interventions [134].

Overall, oxidative stress reduction appears to be closely interconnected with microbiota restoration, SCFA production, and suppression of inflammatory signaling pathways, rather than an independent anti-tumor mechanism.

**Figure 4:**
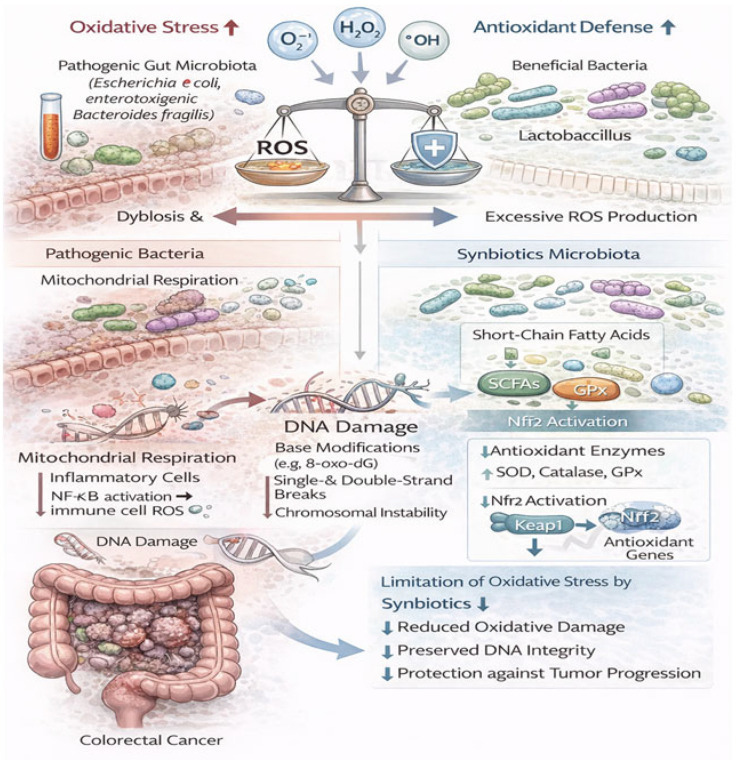
Synbiotic regulation of oxidative stress and DNA damage in CRC. Dysbiosis and pathogenic gut microbiota, including *Escherichia coli* and *Bacteroides fragilis*, contribute to increased production of ROS, such as O_2_^−^, H_2_O_2_, and ^•^OH. These processes are associated with mitochondrial respiration and inflammatory cell activity, leading to DNA damage characterized by base modifications (e.g., 8-oxo-dG), single- and double-strand breaks, and chromosomal instability, ultimately contributing to CRC development. Synbiotics—through beneficial bacteria such as *Lactobacillus*—promote a balanced microbiota and the production of SCFAs. These metabolites are associated with activation of antioxidant pathways, including Nrf2 signaling, and increased activity of antioxidant enzymes such as SOD, catalase, and glutathione peroxidase (GPx). As a result, oxidative stress is reduced, DNA integrity is preserved, and tumor progression is limited. This figure represents a conceptual schematic model summarizing the mechanisms discussed in the text and is intended for illustrative purposes only. Figures were created using BioRender.com and adapted by the authors for the purposes of this review.

Figure 4 illustrates the balance between oxidative stress and antioxidant defense in CRC, highlighting the modulatory role of synbiotics in restoring redox homeostasis.

### 5.2. Molecular Mechanisms

Although these functional effects are critical, they converge at the level of intracellular signaling. The modulation of microbiota composition, metabolites, and immune responses ultimately influences key oncogenic pathways that regulate tumor behavior. The following sections detail how synbiotics interact with major molecular pathways in CRC.

Among the molecular pathways involved in CRC, NF-κB signaling represents a central inflammatory hub linking microbiota dysbiosis, immune activation, and tumor-promoting signaling. In parallel, Wnt/β-catenin and PI3K/Akt/mTOR pathways function primarily as downstream regulators of proliferation, survival, and metabolic reprogramming. Although discussed separately for clarity, these pathways are highly interconnected and collectively mediate the anti-tumor effects of synbiotics.

#### 5.2.1. Wnt/β-Catenin Signaling Pathway

The Wnt/β-catenin signaling pathway represents a fundamental driver of colorectal carcinogenesis and is constitutively activated in the majority of CRC cases, most commonly due to mutations in the APC gene or β-catenin itself [135]. In the context of synbiotic activity, modulation of Wnt/β-catenin signaling appears to occur predominantly as a downstream consequence of microbiota-derived metabolites and reduced inflammatory signaling.

Under physiological conditions, cytoplasmic β-catenin levels are tightly controlled by a multiprotein destruction complex composed of APC, axin, glycogen synthase kinase-3β (GSK-3β), and casein kinase 1 (CK1). This complex promotes phosphorylation of β-catenin, leading to its ubiquitination and subsequent proteasomal degradation, thereby preventing uncontrolled transcriptional activity. Upon activation of Wnt signaling, binding of Wnt ligands to Frizzled receptors and LRP5/6 co-receptors disrupts the destruction complex, allowing β-catenin to accumulate in the cytoplasm and translocate into the nucleus. There, β-catenin interacts with T-cell Factor/Lymphoid Enhancer Factor (TCF/LEF) transcription factors to induce the expression of genes involved in proliferation, survival, and invasion, including c-Myc, cyclin D1, and matrix metalloproteinases (MMPs), thereby promoting tumor growth and progression [138,139,140,141].

Synbiotics modulate this pathway primarily through the production of SCFAs, particularly butyrate. As a HDAC inhibitor, butyrate induces epigenetic modifications that alter chromatin accessibility and gene transcription. In CRC cells, butyrate accumulation—favored by the altered metabolic phenotype of cancer cells—results in enhanced transcriptional regulation of Wnt target genes in a manner that promotes apoptosis rather than proliferation, a phenomenon often referred to as the “butyrate paradox”. This effect is associated with hyperactivation of Wnt signaling beyond a threshold that triggers pro-apoptotic gene expression, including Bax and p21 [112].

In addition to its epigenetic effects, butyrate has been shown to influence β-catenin stability and localization. Experimental studies suggest that SCFAs can promote β-catenin degradation, inhibit its nuclear translocation, or interfere with its interaction with transcriptional co-activators, thereby reducing oncogenic signaling output [136]. These effects collectively contribute to decreased proliferation and increased differentiation of colorectal epithelial cells.

Furthermore, synbiotic-mediated modulation of the gut microbiota plays an indirect but critical role in regulating Wnt signaling. Dysbiosis-associated inflammation enhances Wnt/β-catenin activity through crosstalk with inflammatory pathways such as NF-κB and STAT3, which can stabilize β-catenin and amplify transcriptional activity. By reducing pro-inflammatory cytokine production and limiting activation of these pathways, synbiotics attenuate inflammation-driven Wnt signaling [16,142].

Additionally, microbiota modulation reduces inflammatory signals that otherwise enhance Wnt activity. This results in decreased β-catenin nuclear accumulation and suppression of oncogenic transcription programs.

Overall, synbiotics exert a multi-level regulatory effect on the Wnt/β-catenin pathway, involving epigenetic modulation, metabolic reprogramming, inflammation control, and microbiota restoration. These combined actions lead to suppression of oncogenic transcription programs, reduced tumor cell proliferation, and enhanced apoptosis, highlighting the importance of synbiotics in targeting key molecular drivers of CRC [143,144,145].

Taken together, modulation of Wnt/β-catenin signaling appears to represent a downstream consequence of broader synbiotic effects on microbiota composition, SCFA production, and inflammatory regulation.

#### 5.2.2. NF-κB Signaling Pathway

The NF-κB signaling pathway represents a central molecular link between chronic inflammation and CRC development. Under physiological conditions, NF-κB is maintained in an inactive state in the cytoplasm through its association with inhibitory proteins, primarily IκBα. Upon stimulation by inflammatory signals—such as microbial components, cytokines, or oxidative stress—the IκB kinase (IKK) complex becomes activated, leading to phosphorylation and degradation of IκBα. This allows NF-κB (typically the p65/p50 heterodimer) to translocate into the nucleus, where it induces transcription of genes involved in inflammation, cell survival, proliferation, and angiogenesis. In CRC, persistent activation of NF-κB results in sustained expression of pro-inflammatory cytokines (e.g., IL-6, TNF-α), anti-apoptotic proteins (e.g., Bcl-2, Bcl-xL), and pro-angiogenic factors such as VEGF. This creates a tumor-promoting microenvironment characterized by chronic inflammation, enhanced cell survival, and resistance to apoptosis. Additionally, NF-κB signaling contributes to the activation of other oncogenic pathways, including STAT3 and PI3K/Akt, further amplifying tumor progression [142,143,146,147,148].

Synbiotics modulate NF-κB signaling through several complementary mechanisms. One of the primary effects is the reduction in lipopolysaccharide (LPS)-producing Gram-negative bacteria, which decreases activation of pattern recognition receptors and downstream inflammatory signaling. By restoring microbiota balance, synbiotics limit the upstream triggers of NF-κB activation [134,149]. At the molecular level, SCFAs, particularly butyrate, play a crucial role in inhibiting NF-κB activity. Butyrate acts as a HDAC inhibitor, leading to epigenetic modifications that suppress transcription of NF-κB target genes [150,151]. Furthermore, SCFAs stabilize IκBα, preventing its degradation and thereby blocking NF-κB nuclear translocation. This results in reduced expression of pro-inflammatory mediators and attenuation of inflammation-driven carcinogenesis [113,142].

In addition to direct inhibition, synbiotics indirectly suppress NF-κB signaling by reducing oxidative stress and inflammatory mediator production. ROS are known to activate NF-κB through stimulation of the IKK complex; thus, synbiotic-induced enhancement of antioxidant defenses contributes to further downregulation of this pathway [58,113,152,153].

Emerging evidence indicates that microbiota-derived metabolites can significantly reduce NF-κB activity and tumor burden in experimental models of CRC. These findings highlight the capacity of synbiotics to disrupt the positive feedback loop between inflammation and cancer progression, ultimately shifting the tumor microenvironment toward an anti-inflammatory and anti-tumor state [154].

Overall, synbiotics exert a multi-level inhibitory effect on NF-κB signaling by targeting both upstream activators and downstream transcriptional outputs. This integrated regulation leads to decreased cytokine production, reduced tumor cell survival, and enhanced sensitivity to apoptosis, underscoring the therapeutic potential of synbiotics in inflammation-associated CRC [16,40,68,113,155].

Because NF-κB integrates microbial, inflammatory, and oxidative signals, it may represent one of the central mechanistic hubs through which synbiotics exert anti-tumor effects in CRC.

#### 5.2.3. PI3K/Akt/mTOR Signaling Pathway

Compared with NF-κB-mediated inflammatory signaling, the PI3K/Akt/mTOR pathway mainly reflects downstream effects on cellular metabolism, proliferation, and survival. The PI3K/Akt/mTOR signaling pathway is a central regulator of cell survival, metabolism, growth, and proliferation and is frequently dysregulated in CRC [156]. Activation of phosphatidylinositol 3-kinase leads to the generation of phosphatidylinositol-3,4,5-triphosphate (PIP3), which recruits and activates Akt. Activated Akt subsequently phosphorylates multiple downstream targets, including the mammalian target of rapamycin (mTOR), a key regulator of protein synthesis, cell growth, and metabolic activity. Persistent activation of this pathway promotes tumor progression by enhancing cell proliferation, inhibiting apoptosis, and supporting metabolic reprogramming [113,157].

In CRC, aberrant PI3K/Akt/mTOR signaling is often associated with mutations in PIK3CA, loss of Phosphatase and TENsin homolog (PTEN) protein function, or activation of upstream growth factor receptors. This results in increased tumor cell survival, resistance to apoptosis, and adaptation to metabolic stress. Furthermore, this pathway interacts with other oncogenic signaling networks, including Wnt/β-catenin and NF-κB, amplifying tumor-promoting effects [138,143,156].

Synbiotics modulate the PI3K/Akt/mTOR pathway through multiple interconnected mechanisms. One important effect is the reduction in oxidative stress, which is known to activate PI3K/Akt signaling through redox-sensitive mechanisms. By enhancing antioxidant defenses and decreasing ROS levels, synbiotics indirectly attenuate Akt activation and downstream signaling [113,152].

In addition, synbiotics reduce inflammatory mediator production, thereby limiting activation of upstream receptors that stimulate the PI3K pathway. Chronic inflammation contributes to sustained activation of PI3K/Akt signaling; thus, its suppression by synbiotics leads to decreased proliferative and survival signals [142,143].

A key mechanism involves metabolic reprogramming mediated by SCFAs, particularly butyrate. SCFAs influence cellular energy metabolism and act as signaling molecules that regulate key metabolic pathways. Notably, SCFAs activate AMP-activated protein kinase (AMPK), an energy sensor that negatively regulates mTOR activity. Activation of AMPK inhibits mTOR signaling, leading to reduced protein synthesis, decreased cell growth, and induction of autophagy [113,157].

Furthermore, SCFAs can modulate Akt phosphorylation and interfere with downstream signaling cascades, contributing to reduced tumor cell proliferation and increased apoptosis. This metabolic shift counteracts the anabolic and pro-survival effects of PI3K/Akt/mTOR activation in CRC cells [22,101,113].

Overall, synbiotics exert a multi-level regulatory effect on the PI3K/Akt/mTOR pathway by targeting oxidative stress, inflammation, and metabolic signaling. These combined effects lead to inhibition of tumor cell growth, restoration of apoptosis, and suppression of metabolic adaptations that support cancer progression, highlighting the importance of synbiotics in modulating key oncogenic pathways in CRC [113,143,155,156].

Overall, regulation of PI3K/Akt/mTOR signaling likely reflects secondary metabolic and survival-related effects that occur downstream of microbiota and inflammatory modulation.

#### 5.2.4. Crosstalk Between Pathways

A defining feature of CRC is the extensive crosstalk between major oncogenic signaling pathways, which function as an integrated network rather than independent cascades. In this context, NF-κB signaling can enhance Wnt/β-catenin activity through transcriptional cooperation and inflammatory mediator production, while the PI3K/Akt pathway contributes to the stabilization and nuclear accumulation of β-catenin. In parallel, pro-inflammatory cytokines such as IL-6 and TNF-α are capable of activating multiple signaling pathways simultaneously, further reinforcing proliferative, survival, and metastatic processes. This intricate signaling interplay amplifies tumor progression, promotes resistance to apoptosis, and complicates therapeutic targeting strategies [158,159,160].

Synbiotics exert multi-target regulatory effects by simultaneously modulating these interconnected pathways. Through microbiota restoration and the production of bioactive metabolites such as SCFAs, synbiotics can attenuate aberrant Wnt/β-catenin signaling, suppress NF-κB-mediated inflammation, and inhibit PI3K/Akt-driven survival pathways. This coordinated modulation disrupts the signaling network that sustains tumor growth and enhances cellular homeostasis [159,160].

Importantly, this integrated regulatory capacity represents a significant advantage over single-target therapies, which often fail due to compensatory activation of alternative pathways. By acting at multiple levels within the tumor microenvironment and intracellular signaling networks, synbiotics offer a more comprehensive strategy for limiting CRC progression and improving therapeutic outcomes [138].

**Figure 5:**
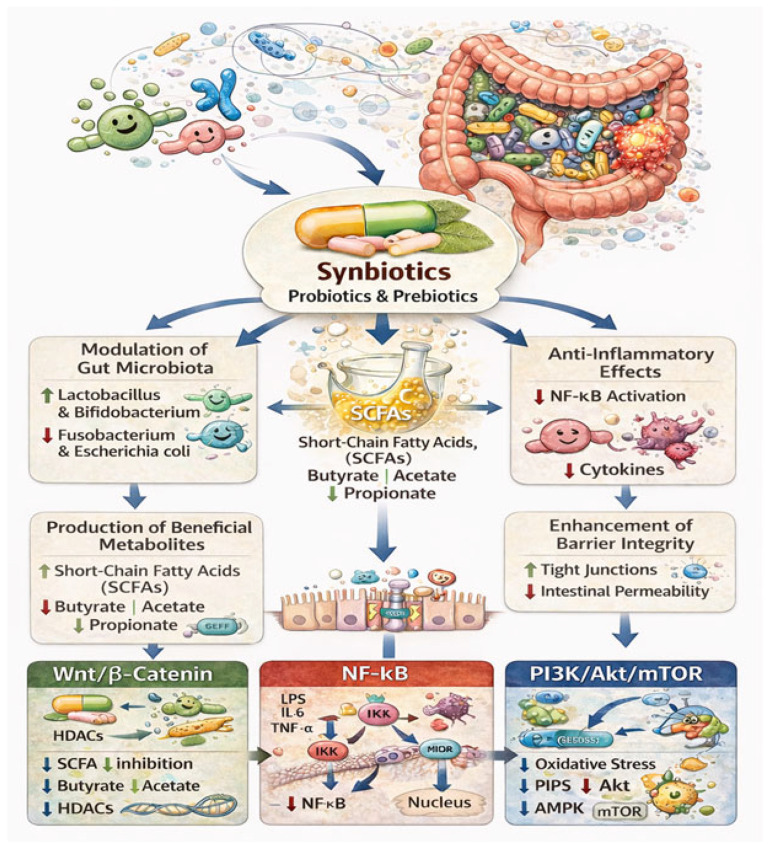
Mechanisms of Action of Synbiotics in CRC. Synbiotics modulate gut microbiota and increase the production of SCFAs, particularly butyrate. These effects contribute to reduced inflammation, enhanced intestinal barrier integrity, decreased oxidative stress, and regulation of cell proliferation. Collectively, these mechanisms influence key oncogenic signaling pathways (Wnt/β-catenin, NF-κB, and PI3K/Akt/mTOR), leading to inhibition of tumor progression and promotion of apoptosis in CRC. This figure represents a conceptual schematic model summarizing the mechanisms discussed in the text and is intended for illustrative purposes only. Figures were created using BioRender.com and adapted by the authors for the purposes of this review.

Figure 5 illustrated how synbiotics modulate gut microbiota and also associated molecular mechanisms, leading to reduced inflammation, decreased oxidative stress, and inhibition of CRC progression.

#### 5.2.5. Evidence from Preclinical and Clinical Studies

Preclinical studies provide compelling evidence supporting the anti-cancer effects of synbiotics in CRC, offering mechanistic insights into their role in modulating tumor biology. Both in vitro experiments and animal models consistently demonstrate that synbiotics exert multi-level effects through microbiota modulation, production of bioactive metabolites, regulation of inflammatory and oxidative pathways, and interference with key oncogenic signaling cascades [161,162,163].

In vitro studies using CRC cell lines such as HCT116, HT-29, and Caco-2 have shown that synbiotic-derived metabolites, particularly SCFAs, play a central role in inhibiting tumor cell proliferation and inducing apoptosis. Butyrate, one of the most extensively studied SCFAs, acts as a HDAC inhibitor, leading to epigenetic reprogramming and modulation of gene expression. These effects result in increased expression of pro-apoptotic genes and suppression of proliferation-associated genes, ultimately reducing tumor cell viability [112,145]. Moreover, SCFAs have been shown to interfere with major oncogenic pathways, including Wnt/β-catenin and PI3K/Akt, by reducing β-catenin nuclear translocation and inhibiting Akt phosphorylation. Moreover, synbiotic metabolites play a crucial role in suppressing inflammatory signaling. In vitro studies have demonstrated that SCFAs inhibit NF-κB activation and reduce the production of pro-inflammatory cytokines, including IL-6 and TNF-α, thereby disrupting inflammation-driven carcinogenesis. These findings highlight the importance of synbiotics in modulating both cellular and molecular mechanisms associated with CRC progression [155].

Animal models, particularly azoxymethane/dextran sodium sulfate (AOM/DSS)-induced CRC models, provide strong in vivo evidence for the protective effects of synbiotics. These studies demonstrate that synbiotic supplementation significantly reduces tumor incidence, tumor size, and histological severity while improving overall intestinal homeostasis. A key mechanism underlying these effects is the modulation of gut microbiota composition, characterized by an increase in beneficial bacteria such as *Lactobacillus* and *Bifidobacterium* and a reduction in pathogenic species associated with carcinogenesis [164].

More recent studies (2024–2026) further strengthen this evidence by demonstrating that specific probiotic strains and synbiotic formulations exert targeted anti-cancer effects through multiple mechanisms. For instance, administration of probiotic mixtures in AOM/DSS models has been shown to significantly reduce tumor burden and enhance apoptosis through p53-dependent mechanisms [165]. Similarly, *Bifidobacterium longum* has been reported to suppress colorectal tumor development by restoring microbiota balance and enhancing anti-tumor immune responses [166]. Other studies have demonstrated that *Lactobacillus rhamnosus* reduces pro-inflammatory cytokines such as IL-6 and TNF-α and inhibits NF-κB signaling, thereby attenuating inflammation-driven carcinogenesis [167]. Other studies have reported that *Lactobacillus casei* reduces tumor number and size in AOM/DSS models while promoting apoptosis through activation of caspase pathways and suppression of inflammatory mediators. In addition, *Lacticaseibacillus rhamnosus* has been shown to significantly reduce levels of IL-1β, IL-6, TNF-α, and NF-κB, indicating strong anti-inflammatory effects in CRC models. Furthermore, *Limosilactobacillus fermentum* GR-3 has been reported to exert antioxidant effects, reduce oxidative stress, and improve intestinal barrier integrity, leading to decreased tumor burden and restoration of colon morphology. Recent investigations also highlight that probiotics can suppress tumor growth and prevent CRC development by modulating microbiota composition and reducing inflammation in murine models [168]. More recently, *Lactobacillus plantarum* Zhang-LL was found to remodel gut microbiota composition and alter tumor-associated metabolic pathways, leading to suppression of CRC progression [169]. Collectively, these studies highlight that synbiotics exert anti-tumor effects through a combination of microbiome modulation, metabolic reprogramming, inflammation suppression, and oxidative stress reduction. These mechanisms converge to inhibit tumor cell proliferation, promote apoptosis, and reshape the tumor microenvironment into a less permissive state for cancer progression [170].

Table 3 illustrates the results of preclinical studies on synbiotics’ role in CRC.

Clinical evidence on synbiotics in CRC has grown substantially over the past decade, with increasing numbers of randomized controlled trials (RCTs), cohort studies, and meta-analyses investigating their role as adjunctive therapies. Although still heterogeneous, current data suggest that synbiotics exert clinically relevant effects on postoperative outcomes, chemotherapy-associated toxicity, and gut microbiota composition, primarily through modulation of inflammation, barrier function, and microbial homeostasis [174].

Clinical trials evaluating synbiotics in CRC patients have largely focused on three main contexts: perioperative administration, supportive care during chemotherapy or radiotherapy, and microbiome-targeted interventions in high-risk populations. Early studies established that probiotics and synbiotics could reduce postoperative infections and improve recovery following colorectal surgery. More recent investigations have expanded these findings to include modulation of systemic inflammation, enhancement of immune responses, and improvement of treatment tolerance [16,91,92].

A growing number of meta-analyses have confirmed that microbiota-targeted therapies significantly improve clinical outcomes in CRC patients. For example, a comprehensive meta-analysis including over 1000 patients demonstrated that probiotic and synbiotic supplementation significantly reduced postoperative complications, including surgical site infections and sepsis, while also shortening hospital stay [96].

Recent studies also suggest that synbiotics may influence oncologic outcomes indirectly by modulating tumor-associated inflammation and metabolic pathways, although long-term survival data remain limited [175].

The strongest clinical evidence for synbiotics in CRC is observed in the perioperative setting. Multiple randomized controlled trials have demonstrated that synbiotic supplementation significantly reduces postoperative infectious complications and improves recovery.

For instance, a landmark randomized controlled trial by Zhang et al. showed that preoperative administration of probiotics significantly reduced postoperative infections in CRC patients undergoing surgery. Similarly, Yang et al. reported that perioperative probiotic treatment decreased infection rates and improved immune parameters, including increased levels of IgA and reduced inflammatory cytokines [176].

More recent studies reinforce these findings. A meta-analysis by Gao et al. (2025), including multiple RCTs, demonstrated that synbiotics reduced postoperative infection risk by approximately 40–50%, along with improvements in intestinal barrier integrity and immune response [96].

In addition, synbiotics have been shown to reduce bacterial translocation and endotoxemia by strengthening tight junctions and limiting permeability, thereby decreasing systemic inflammatory responses [137]. These effects contribute to reduced rates of complications such as anastomotic leakage, sepsis, and prolonged hospitalization.

Chemotherapy-related gastrointestinal toxicity, particularly diarrhea, mucositis, and dysbiosis, represents a major clinical challenge in CRC management. Emerging evidence suggests that synbiotics may play a role in mitigating these adverse effects [177].

A recent systematic review and meta-analysis by Yao et al. (2025) [90] evaluated the efficacy of probiotics and synbiotics in patients undergoing chemotherapy for gastrointestinal cancers. The study reported that supplementation significantly reduced the incidence and severity of chemotherapy-induced diarrhea and improved overall treatment tolerance [90].

Similarly, randomized clinical trials have shown that probiotics can reduce chemotherapy-associated gastrointestinal toxicity by stabilizing gut microbiota and maintaining mucosal integrity. For example, patients receiving probiotic supplementation during chemotherapy exhibited lower rates of diarrhea and improved quality of life compared to control groups [178].

Importantly, several clinical studies and meta-analyses indicate that synbiotics may reduce chemotherapy-related gastrointestinal adverse events, particularly diarrhea and mucositis, thereby improving treatment tolerance and patient adherence. Recent analyses reported reductions in chemotherapy-associated gastrointestinal complications ranging from approximately 20% to 50%, depending on the probiotic formulation, cancer type, and treatment regimen [90,96]. However, although synbiotics appear to improve tolerability and reduce treatment interruptions, current evidence regarding direct enhancement of chemotherapy efficacy, tumor response, progression-free survival, or overall survival remains limited and heterogeneous.

However, results remain inconsistent. Some studies report only modest benefits or no statistically significant improvements, likely due to differences in probiotic strains, dosing regimens, and patient populations [179]. Nonetheless, the overall trend supports a protective role of synbiotics against treatment-related toxicity.

The current clinical evidence remains highly heterogeneous, which represents a major limitation in interpreting the efficacy of synbiotics in CRC management. Considerable variability exists among studies regarding probiotic strains, synbiotic formulations, dosing regimens, treatment duration, patient populations, and evaluated clinical endpoints. Some studies used single-strain probiotics, whereas others investigated multi-strain formulations combined with different prebiotic substrates, making direct comparisons difficult. In addition, endpoints ranged from postoperative infection rates and chemotherapy-induced diarrhea to microbiota composition and inflammatory biomarkers. This heterogeneity likely contributes to the variability in reported outcomes and currently limits the establishment of standardized synbiotic protocols for CRC patients.

One of the most consistent findings across clinical studies is the ability of synbiotics to modulate gut microbiota composition. Dysbiosis is a hallmark of CRC and is associated with increased inflammation, carcinogen production, and tumor progression.

Randomized controlled trials have demonstrated that synbiotic supplementation increases microbial diversity and promotes beneficial bacteria such as *Lactobacillus* and *Bifidobacterium*, while reducing pathogenic species including *Fusobacterium nucleatum* [22].

For example, a double-blind RCT by Meng et al. (2025) showed that prebiotic supplementation significantly improved gut microbiota composition and reduced inflammatory markers in patients with colorectal adenomas [180]. Similarly, synbiotic interventions have been shown to alter mucosa-associated microbiota and reduce pro-inflammatory signaling in patients undergoing radiotherapy [181].

These microbiome changes are associated with increased production of SCFAs, improved barrier function, and reduced systemic inflammation, all of which contribute to a less tumor-promoting environment.

To provide a more comprehensive overview of current clinical evidence, additional comparative information regarding adverse events, treatment tolerance, therapeutic outcomes, and microbiota-related effects has been incorporated into Table 4.

#### 5.2.6. Synbiotics as Adjunct Therapy in CRC Management

The integration of synbiotics as adjunctive therapy in CRC management has gained increasing attention due to their capacity to modulate the gut microbiota, regulate immune responses, and influence treatment-related outcomes. Emerging evidence suggests that synbiotics may enhance the efficacy of conventional therapies, including chemotherapy and radiotherapy, while simultaneously reducing treatment-related toxicity and improving patient recovery and quality of life.

The combination of synbiotics with standard oncologic treatments such as chemotherapy and radiotherapy represents a promising therapeutic strategy. These treatments, while effective in targeting tumor cells, often disrupt gut microbiota composition and intestinal barrier integrity, leading to dysbiosis and increased susceptibility to inflammation and toxicity.

Synbiotics have been shown to counteract these effects by restoring microbial balance and maintaining intestinal homeostasis. In clinical and translational studies, synbiotic supplementation has been associated with preservation of beneficial bacteria, increased production of SCFAs, and improved mucosal integrity in patients undergoing chemotherapy or radiotherapy [16,134].

For example, synbiotics have been reported to reduce radiation-induced intestinal injury by modulating inflammatory pathways and promoting epithelial repair. This effect is largely attributed to SCFA-mediated mechanisms, including activation of anti-inflammatory pathways and enhancement of epithelial regeneration [181].

In addition to mitigating adverse effects, synbiotics may enhance the efficacy of cancer therapies. The gut microbiota has been increasingly recognized as a critical modulator of treatment response, influencing both chemotherapy and immunotherapy outcomes. Synbiotics can improve treatment efficacy through several mechanisms. First, they modulate immune responses by promoting anti-inflammatory cytokines and enhancing immune cell function, which may support anti-tumor activity. Second, microbial metabolites such as butyrate can influence tumor cell sensitivity to chemotherapy by modulating gene expression and apoptosis pathways [101].

Recent studies suggest that microbiota-targeted interventions may improve responsiveness to cancer therapies by reducing systemic inflammation and altering the tumor microenvironment. These effects highlight the potential role of synbiotics in optimizing therapeutic outcomes and overcoming resistance mechanisms [175].

One of the most clinically relevant benefits of synbiotics is their ability to reduce treatment-related toxicity, particularly gastrointestinal side effects. Chemotherapy-induced diarrhea (CID) and mucositis are common complications in CRC patients, often leading to dose reduction or discontinuation of therapy. Clinical trials and meta-analyses have shown that synbiotics and probiotics can significantly reduce the incidence and severity of diarrhea in patients undergoing chemotherapy [90]. This protective effect is mediated by stabilization of gut microbiota, enhancement of barrier function, and suppression of inflammation.

Additionally, synbiotics have been associated with reduced mucosal damage and improved epithelial repair, thereby alleviating mucositis and other gastrointestinal symptoms. These effects are linked to increased SCFA production and modulation of inflammatory pathways such as NF-κB [90,113].

However, it should be noted that the magnitude of these benefits varies across studies, and further large-scale clinical trials are needed to confirm their efficacy.

Synbiotics also play an important role in postoperative recovery and overall quality of life in CRC patients. By improving gut microbiota composition and reducing inflammation, synbiotics contribute to faster recovery following surgery and reduced postoperative complications. Clinical studies have demonstrated that synbiotic supplementation can improve gastrointestinal function, reduce infection rates, and shorten hospital stay [176]. These benefits are closely associated with enhanced immune responses and improved barrier integrity.

Furthermore, synbiotics may positively influence patient-reported outcomes, including quality of life, by reducing gastrointestinal symptoms, improving nutrient absorption, and stabilizing metabolic homeostasis. Patients receiving synbiotic supplementation often report reduced fatigue, improved appetite, and better overall well-being during and after treatment [92].

## 6. Future Perspectives

The growing understanding of the gut microbiome in CRC has paved the way for the development of personalized therapeutic strategies, in which synbiotics are tailored to individual patient characteristics. Advances in microbiome research, combined with emerging technologies such as metagenomics and metabolomics, are transforming synbiotics from generalized interventions into precision medicine tools capable of targeting specific microbial and molecular profiles.

One of the most promising directions in CRC research is the integration of microbiome profiling into precision oncology. Recent studies have demonstrated that CRC patients exhibit distinct microbial signatures characterized by enrichment of pro-inflammatory and genotoxic bacteria and depletion of beneficial commensals [93,175]. These microbial patterns are not only associated with tumor development but also influence treatment response and disease progression.

Despite the promising preclinical and clinical evidence, several challenges limit the effective translation of synbiotics into routine clinical practice in CRC. One of the major limitations is the marked heterogeneity in synbiotic formulations, including differences in probiotic strains, prebiotic substrates, and their combinations, which complicates cross-study comparisons and limits reproducibility [21,40]. In addition, substantial variability exists in dosing regimens, with studies employing a wide range of colony-forming units (CFU) without standardized dose–response relationships [73]. Treatment duration also varies considerably, ranging from short-term perioperative administration to long-term supplementation, further contributing to inconsistent clinical outcomes [90].

Another critical challenge is the high inter-individual variability in gut microbiota composition, which significantly influences patient response to synbiotic interventions. Factors such as diet, antibiotic exposure, host genetics, and environmental influences shape microbiome structure and function, thereby affecting therapeutic efficacy [40,93]. Furthermore, the clinical translation of synbiotics is hindered by the limited number of large-scale, well-designed randomized controlled trials and the lack of standardized regulatory frameworks governing synbiotic products [21,175]. Issues related to strain viability, product quality, and patient adherence also represent important barriers to implementation in clinical settings [73]. Future studies should aim to standardize synbiotic formulations and treatment protocols while accounting for patient-specific microbiome profiles to improve reproducibility and clinical applicability.

Addressing these challenges will be essential for integrating synbiotics into precision medicine strategies in CRC, enabling the development of standardized, evidence-based, and personalized therapeutic approaches.

Metagenomic analyses enable the identification of these signatures at high resolution, allowing stratification of patients based on microbiome composition. For example, large-scale microbiome profiling studies have shown that specific microbial clusters can distinguish CRC patients from healthy individuals and may serve as biomarkers for early detection and prognosis [184]. In this context, synbiotics can be integrated into precision medicine frameworks by targeting patient-specific dysbiosis.

From a translational perspective, the integration of precision microbiome approaches represents a critical step toward the clinical implementation of synbiotics in CRC. Advances in microbiome profiling technologies, including metagenomics and metabolomics, enable the identification of patient-specific microbial signatures associated with tumor development and treatment response. In parallel, host genetic factors, such as polymorphisms in immune regulation and metabolic pathways, may influence both microbiome composition and responsiveness to synbiotic interventions. The integration of these datasets through artificial intelligence (AI) and machine learning approaches offers the potential to design personalized synbiotic formulations tailored to individual microbiome profiles and host characteristics. Such strategies could optimize therapeutic efficacy, improve patient stratification, and reduce variability in clinical outcomes, aligning synbiotic-based interventions with the principles of precision medicine.

Recent evidence also highlights the oral and salivary microbiome as an emerging non-invasive biomarker in CRC. Oral dysbiosis and oral–gut microbial translocation have been increasingly implicated in colorectal carcinogenesis, particularly through oral-associated bacteria such as *Fusobacterium nucleatum*, which has been detected in colorectal tumor tissues and linked to tumor progression [185,186,187]. In addition, salivary microbiome profiling has been proposed as a minimally invasive approach for CRC risk assessment, early detection, and monitoring of treatment-associated dysbiosis [188]. Although the present review primarily focuses on the gut microbiota, integration of oral–gut microbiome interactions may further expand future translational and precision medicine approaches in CRC.

The concept of personalized synbiotic formulations represents a major shift from traditional approaches. Inter-individual variability in microbiota composition, genetics, diet, and environmental exposures significantly influences the efficacy of synbiotics. This variability underscores the need for patient-specific formulations designed to enhance colonization of beneficial microbes and optimize metabolic outputs such as SCFA production.

Emerging approaches involve the use of computational modeling and microbiome-informed design to predict how specific probiotic strains and prebiotic substrates interact within an individual’s gut ecosystem. Furthermore, the field of pharmacomicrobiomics highlights the role of microbiota in drug metabolism and response, suggesting that synbiotics could be tailored to improve drug efficacy and reduce toxicity based on individual microbial profiles [189].

Diet and lifestyle are major determinants of gut microbiota composition and play a critical role in the success of synbiotic interventions. Western dietary patterns have been associated with dysbiosis and increased CRC risk, whereas fiber-rich diets promote beneficial microbial communities and SCFA production [91,93].

Future therapeutic strategies increasingly focus on integrating synbiotics with personalized nutrition plans to achieve synergistic effects. Dietary fibers act as prebiotic substrates that enhance probiotic activity, amplifying beneficial outcomes. In addition, lifestyle factors such as physical activity, antibiotic exposure, and environmental influences further shape microbiome composition and should be considered when designing personalized interventions.

Technological advances are central to the future of synbiotic therapy. Metagenomics allows comprehensive analysis of microbial communities at the genetic level, enabling precise characterization of microbiome composition and functional potential [190].

Complementarily, metabolomics provides insights into the functional output of the microbiome by analyzing bioactive metabolites such as SCFAs, bile acids, and other signaling molecules. Integration of metagenomics and metabolomics enables a systems-level understanding of host–microbiome interactions and their role in CRC.

Recent studies emphasize that multi-omics approaches can identify complex networks linking microbiota composition, metabolic activity, and cancer progression, facilitating the discovery of novel biomarkers and therapeutic targets [190]. Additionally, advances in artificial intelligence and machine learning are enhancing the ability to analyze large datasets and predict patient-specific responses to microbiome-targeted therapies [175].

## 7. Conclusions

CRC represents a complex and multifactorial disease in which interactions between the gut microbiota, host immune system, metabolic processes, and intracellular signaling pathways play a central role in tumor initiation and progression. The evidence presented throughout this work highlights synbiotics as promising modulators of these interconnected systems, capable of influencing both the tumor microenvironment and key molecular mechanisms involved in carcinogenesis.

At the functional level, synbiotics exert beneficial effects through restoration of microbial balance, increased production of SCFAs, enhancement of intestinal barrier integrity, and reduction in chronic inflammation and oxidative stress. These effects translate into decreased DNA damage, improved immune regulation, and suppression of pro-tumorigenic processes.

Importantly, these functional mechanisms converge at the level of intracellular signaling pathways. Synbiotics have been shown to modulate major oncogenic pathways, including Wnt/β-catenin, NF-κB, and PI3K/Akt/mTOR, which are critical regulators of cell proliferation, survival, inflammation, and metabolism in CRC. Through this multi-target regulation, synbiotics disrupt the molecular crosstalk that drives tumor progression, representing a significant advantage over single-target therapeutic approaches.

The integration of microbiome research into precision medicine represents a key future direction. Advances in metagenomics, metabolomics, and computational modeling are expected to enable the development of personalized synbiotic interventions tailored to individual patient profiles.

Overall, synbiotics emerge as a promising adjunctive strategy in CRC, bridging the gap between microbiota modulation and molecular oncology. Their ability to simultaneously target multiple biological processes positions them as valuable components of future integrative and personalized therapeutic approaches.

Recent evidence remains limited by substantial heterogeneity among clinical studies, and several important questions remain unresolved. Key unanswered clinical issues include the optimal synbiotic formulations and dosing regimens, duration of administration, patient selection criteria, long-term safety, and the extent to which synbiotics can improve therapeutic response and survival outcomes in CRC patients. In addition, the identification of microbiome-based predictive biomarkers and the integration of personalized synbiotic strategies into standard oncologic care require further large-scale, well-controlled clinical trials.

Further research is required to standardize formulations, optimize clinical protocols, and fully elucidate their long-term impact on cancer outcomes.

## Figures and Tables

**Table 1:** Individual and synergistic effects of microbiota in CRC.

Category	Bacterium/Microbial State	Immune Mechanism	Molecular Pathway/Target	Effect on Tumor Development	References (Author, Year)
Individual species	*Fusobacterium nucleatum*	Suppression of anti-tumor immunity	Fap2–TIGIT interaction; ↓ NK cells; ↓ CD8^+^ T cells; ↓ IFN-γ	Immune evasion; tumor progression	Gur et al., 2015 [45]; Kostic et al., 2013 [17]
Individual species	*Bacteroides fragilis* (ETBF)	Pro-inflammatory (Th17 response)	↑ IL-17; activation of NF-κB and STAT3	Chronic inflammation; angiogenesis; tumor growth	Wu et al., 2009 [46]; Chung et al., 2018 [48]
Individual species	pks^+^ *Escherichia coli*	Immune + genotoxic effects	Colibactin; DNA damage; inflammatory signaling	Mutation accumulation; tumor initiation	Zhang et al., 2024 [52]; Chen et al., 2023 [54]
Individual species	*Peptostreptococcus anaerobius*	Innate immune activation	TLR signaling; PI3K/Akt; ROS production	Oxidative stress; tumor proliferation	Long et al., 2019 [56]
Microbial imbalance	Dysbiotic microbiota	Expansion of immunosuppressive cells	↑ Tregs; ↑ MDSCs; ↑ IL-10; ↑ TGF-β	Suppressed anti-tumor immunity; immune escape	Karam et al., 2025 [40]
Microbial imbalance	Dysbiotic microbiota	Impaired antigen presentation	Dysfunction of dendritic cells; altered cytokine signaling	Reduced T cell activation; tumor tolerance	Karam et al., 2025 [40] Grellier et al., 2024 [41]
Synergistic effect	Multiple bacterial species (microbial consortium)	Combined immune dysregulation	NF-κB/STAT3 activation; IL-6, IL-17 signaling	Chronic inflammation; immune evasion; tumor progression	Grellier et al., 2024 [41]; Grivennikov et al., 2010 [68]

TIGIT = T cell immunoreceptor with Ig and ITIM domains; IFN-γ = interferon-γ; NK = natural killer; IL17 = interleukin-17; STAT3 = signal transducer and activator of transcription 3; NF-κB = nuclear factor kappa B; TLR = toll like-receptor; PI3K = phosphoinositide 3-kinase; ROS = reactive oxygen species; Tregs = regulatory T cells; IL-10 = interleukin-10; TGF-β = transforming growth factor beta; IL-6 = interleukin-6.

**Table 2:** Synbiotic combinations and their molecular mechanisms in CRC and gut homeostasis.

Synbiotic Combination	Components (Probiotic + Prebiotic)	Molecular Mechanisms	Biological Effects	Clinical Relevance	References
*Bifidobacterium longum* + FOS/inulin	*Bifidobacterium* spp. + fructooligosaccharides	↑ SCFA (butyrate) production; HDAC inhibition; activation of GPR41/43 receptors	Improved epithelial barrier; anti-inflammatory effects	CRC prevention; reduced inflammation	Moreira, 2024 [74] He, 2025 [73]
*Lactobacillus rhamnosus* + GOS	*Lactobacillus* spp. + galactooligosaccharides	Modulation of NF-κB signaling; ↑ tight junction proteins; ↓ pro-inflammatory cytokines (TNF-α, IL-6)	Enhanced gut integrity; immune modulation	Reduced intestinal inflammation	Al-Habsi, 2024 [76] Smolinska, 2025 [71]
Multi-strain synbiotic	*Lactobacillus* + *Bifidobacterium* + *S. thermophilus* + inulin	Microbiota diversification; ↑ SCFAs; modulation of IL-6/STAT3 pathway	Restoration of microbial balance; reduced dysbiosis	Improved gut homeostasis	Kezer, 2025 [87] Gao, 2025 [96]
*Bifidobacterium lactis* + resistant starch	*B. lactis* + resistant starch	↑ butyrate → apoptosis induction; cell cycle arrest; HDAC inhibition	Anti-tumor effects; reduced epithelial proliferation	CRC prevention potential	Moreira, 2024 [74] He, 2025 [73]
Synbiotics in CRC therapy	Mixed strains + dietary fibers	↓ NF-κB activation; ↓ IL-6/STAT3 signaling; ↑ Treg differentiation; ↓ Th17 response	Reduced inflammation; immune balance restoration	Improved chemotherapy tolerance; reduced complications	Gao, 2025 [96] He, 2025 [73] Yao, 2025 [90]
Next-generation synbiotics	Targeted strains + precision prebiotics	Specific metabolic pathway targeting; modulation of microbiome gene expression; improved colonization	Targeted microbiota modulation; metabolic regulation	Personalized CRC therapy	Kezer, 2025 [87] Yao, 2025 [90]

SCFA = short-chain fatty acid; HDAC = histone deacetylase; GPR 41/43 = G protein-coupled receptor 41/43; NF-κB = nuclear factor kappa B; IL-6 = interleukin-6; TNF-α = tumor necrosis factor alfa; STAT3 = signal transducer and activator of transcription 3.

**Table 3:** Preclinical Studies on Synbiotics in CRC.

Study	Model	Intervention	Key Findings
Leung HKM et al., 2024 [165]	AOM/DSS mice	Probiotic mixture	↓ tumorigenesis, ↓ tumor burden, ↑ apoptosis (p53), restored microbiota, improved barrier function
Shang F et al., 2024 [166]	Mouse CRC model	*Bifidobacterium longum*	↓ tumor development, ↑ immune response, microbiota restoration
Abdorrashidi M et al., 2025 [171]	AOM/DSS mice & in vitro	*Lactobacillus casei*	↓ tumor size/number, ↑ apoptosis (caspases), ↓ cytokines
Zhang J et al., 2024 [167]	AOM/DSS mice	*Lactobacillus rhamnosus*	↓ IL-6, TNF-α, NF-κB signaling, reduced inflammation
Zhou T et al., 2024 [168]	AOM/DSS mice	*L. fermentum GR-3*	↓ oxidative stress, improved barrier function, ↓ tumor burden
Zhu J et al., 2026 [169]	AOM/DSS mice	*L. plantarum* Zhang-LL	microbiota remodeling, metabolic reprogramming, ↓ CRC progression
Niechcial A et al., 2025 [172]	Murine CRC model	Probiotic formulation	↓ tumor growth, microbiota modulation
Thoda C et al., 2025 [164]	Multiple models	Synbiotic formulations	↓ tumor incidence, ↓ inflammation, ↑ apoptosis
Ma F et al., 2022 [173]	AOM/DSS mice	*Lactiplantibacillus plantarum*-12	↓ colon cancer burden, ↓ p65/p-p65, ↑ IκB-α, ↓ PCNA, ↑ Bax; reduced inflammation and promoted apoptosis

AOM/DSS = azoxymethane/dextran sodium sulfate; IL-6 = interleukin-6; TNF-α = tumor necrosis factor-α; NF-κB = nuclear factor kappa B; CRC = colorectal cancer.

**Table 4:** Clinical Studies on Synbiotics in CRC.

Study	Design	Population	Intervention	Synbiotic Formulation	Dosing	Treatment Duration	Chemotherapy-Related Adverse Events	Chemotherapy Effectiveness	Key Findings
Zhang JW, 2012 [182]	RCT	CRC surgery patients	Probiotics	*Lactobacillus* + *Bifidobacterium* spp.	~10^9^–10^10^ CFU/day	Perioperative (5–7 days pre/post-op)	Not applicable; perioperative study	Not assessed	↓ postoperative infections
Yang Y, 2016 [176]	RCT	CRC patients	Probiotics (perioperative)	Multi-strain probiotics (*Lactobacillus*, *Bifidobacterium*)	~10^9^–10^10^ CFU/day	Perioperative	Not applicable; perioperative study	Not assessed	↓ infections, ↑ immune response
Gao S, 2025 [96]	Meta-analysis	CRC surgery patients	Pro/synbiotics	Heterogeneous formulations across studies	Variable (10^7^–10^11^ CFU/day)	Variable (days to weeks)	Not applicable; mainly surgical outcomes	Not assessed	↓ infections (~40–50%), ↓ hospital stay
Yao B, 2025 [90]	Meta-analysis	GI cancer patients	Pro/synbiotics	Mixed probiotic strains ± prebiotics (FOS, inulin)	Variable	During chemotherapy cycles	↓ chemotherapy-induced diarrhea and gastrointestinal toxicity	Direct anti-tumor efficacy not clearly established; possible improved treatment tolerance/adherence	↓ chemotherapy-induced diarrhea
Meng S, 2025 [180]	Double-blind RCT	Adenoma patients	Prebiotics	FOS/dietary fiber	Not specified	Weeks–months	Not applicable; non-chemotherapy population	Not assessed	↑ microbiota diversity, ↓ inflammation
Stene C, 2025 [181]	RCT	Rectal cancer patients undergoing radiotherapy	Synbiotics	Probiotics + prebiotics (inulin-based)	~10^9^ CFU/day	During radiotherapy	Not chemotherapy-specific; ↓ treatment-related gut injury	Effect on oncologic response not clearly established	↓ gut injury, ↓ inflammation
Kotzampassi, 2015 [183]	RCT	GI cancer surgery patients	Synbiotics	*Lactobacillus* + *Bifidobacterium* + prebiotics	~10^9^–10^10^ CFU/day	Perioperative	Not applicable; perioperative study	Not assessed	↓ postoperative infections, ↓ hospital stay, improved immune response
Chen C, 2025 [175]	Review/clinical data	CRC patients	Microbiota-targeted therapy	Not standardized	Not specified	Not specified	Potential reduction in treatment-related toxicity; evidence heterogeneous	Potential improvement in therapy response through immune and microbiota modulation; clinical evidence limited	Improved immune response, microbiota modulation
Osterlund P, 2007 [178]	RCT	CRC chemotherapy patients	*Lactobacillus rhamnosus* GG	Single strain (LGG)	~10^10^ CFU/day	During chemotherapy	↓ severe chemotherapy-induced diarrhea; improved gastrointestinal tolerance	No clear evidence of increased chemotherapy efficacy; may support treatment continuity	↓ incidence of severe diarrhea, improved treatment tolerance

RCT = randomized controlled trial; FOS = Fructooligosaccharides; CRC = colorectal cancer; GI = gastrointestinal; LGG = Lactobacillus rhamnosus GG; CFU = Colony-Forming Unit.

## Data Availability

No new data were created or analyzed in this study. Data sharing is not applicable to this article.

## Abbreviations

^•^OH	hydroxyl radicals
AI	artificial intelligence
AMPK	AMP-activated protein kinase
AOM	azoxymethane
Bcl-2	B-cell lymphoma 2
Bcl-xL	B-cell lymphoma-extra large
*BFT*	*Bacteroides fragilis* toxin
CFU	Colony-Forming Unit
CK1	casein kinase 1
COX-2	ciclooxigenase 2
CRC	colorectal cancer
DCA	deoxycholic acid
dMMR	mismatch repair-deficient
DSS	dextran sodium sulfate
EMT	epithelial–mesenchymal transition
*ETBF*	*Enterotoxigenic Bacteroides fragilis*
FMT	fecal microbiota transplantation
FOS	fructooligosaccharides
GOS	galactooligosaccharides
GPR 41/43	G protein-coupled receptor 41/43
GSK-3β	glycogen synthase kinase-3β
H_2_O_2_	hydrogen peroxide
H_2_S	hydrogen sulfide
HDACs	histone deacetylases
ICLs	interstrand crosslinks
ICLs	interstrand DNA crosslinks
IFN-γ	Interferon-γ
IkB	inhibitory kappa B proteins
IKK	IκB kinase
IL-17	interleukin-17
IL-6	interleukin-6
IL-1β	interleukin-1β
iNOS	inducible nitric oxide synthase
ISAPP	International Scientific Association for Probiotics and Prebiotics
JAKs	janus kinases
LCA	lithocholic acid
LEF	lymphoid enhancer factor
LGG	*Lactobacillus rhamnosus* GG
LPS	lipopolysaccharide
MAMPs	microbial-associated molecular patterns
MAPK	mitogen-activated protein kinase
MDA	malondialdehyde
MDSCs	myeloid-derived suppressor cells
MOMP	mitochondrial outer membrane permeability
MSI-H	microsatellite instability-high
MSS	microsatellite stable
mTOR	mammalian target of rapamycin
MyD88	myeloid differentiation primary response gene 88 protein
NF-κB	nuclear factor kappa B
NK	natural killer
NLRs	NOD-like receptors
NOD	nucleotide-binding oligomerization domain
Nrf2	nuclear factor erythroid 2-related factor 2
O_2_^−^	superoxide anion
PD-L1	programmed death-ligand 1
PI3K	phosphoinositide 3-kinase
PIP3	phosphatidylinositol-3,4,5-triphosphate
Pks	polyketide synthase
PRP	pattern recognition receptor
PTEN	phosphatase and TENsin homolog
RCTs	randomized controlled trials
RNS	nitrogen species
ROS	reactive oxygen species
SASP	senescence-associated secretory phenotype
SCFAs	short-chain fatty acids
SOD	superoxide dismutase
SREBP2	sterol regulatory element-binding protein 2
STAT3	signal transducer and activator of transcription 3
TCF	T-cell factor
TGF-β	transforming growth factor-β
TIGIT	T cell immunoreceptor with Ig and ITIM domains
TLR4	Toll-like receptor 4
TLRs	Toll-like receptors
TNF-α	tumor necrosis factor alfa
TRAIL	TNF-related apoptosis-inducing ligand
Tregs	regulatory T cells
TRIF	TIR-domain-containing adapter inducing interferon-β
VEGF	vascular endothelial growth factor

## References

[1] Bray F., Laversanne M., Sung H., Ferlay J., Siegel R.L., Soerjomataram I., Jemal A. (2024). Global cancer statistics 2022: GLOBOCAN estimates of incidence and mortality worldwide for 36 cancers in 185 countries. CA Cancer J. Clin..

[2] Sung H., Ferlay J., Siegel R.L., Laversanne M., Soerjomataram I., Jemal A., Bray F. (2021). Global Cancer Statistics 2020: GLOBOCAN estimates of incidence and mortality worldwide for 36 cancers in 185 countries. CA Cancer J. Clin..

[3] Arnold M., Sierra M.S., Laversanne M., Soerjomataram I., Jemal A., Bray F. (2017). Global patterns and trends in colorectal cancer incidence and mortality. Gut.

[4] Dekker E., Tanis P.J., Vleugels J.L.A., Kasi P.M., Wallace M.B. (2019). Colorectal cancer. Lancet.

[5] GBD 2019 Colorectal Cancer Collaborators (2022). Global, regional, and national burden of colorectal cancer and its risk factors, 1990-2019: A systematic analysis for the Global Burden of Disease Study 2019. Lancet Gastroenterol. Hepatol..

[6] Xi Y., Xu P. (2021). Global colorectal cancer burden in 2020 and projections to 2040. Transl. Oncol..

[7] Siegel R.L., Miller K.D., Wagle N.S., Jemal A. (2023). Cancer statistics, 2023. CA Cancer J. Clin..

[8] Mao J.X., Gao R., Wang Y., Yan X.B., Wang H.H. (2025). Surgical treatment of colorectal cancer: A multidimensional review. World J. Gastrointest. Surg..

[9] Kajiwara Y., Ueno H. (2024). Essential updates 2022-2023: Surgical and adjuvant therapies for locally advanced colorectal cancer. Ann. Gastroenterol. Surg..

[10] Oh J.M., Kim S., Tsung C., Kent E., Jain A., Ruff S.M., Zhang H. (2025). Comprehensive review of the resistance mechanisms of colorectal cancer classified by therapy type. Front. Immunol..

[11] Adeleke A., Adebayo A.S., Agbaje K., Olajubutu O., Adesina S.K. (2025). Colorectal cancer: Therapeutic approaches and their complications. Biomedicines.

[12] Colombo A., Gebbia V., Porretto C.M. (2024). Immunotherapy in colorectal cancer: A review. Explor. Res. Hypothesis Med..

[13] Keivany M.R., Shojae E., Besharatloo M., Latifi H., Barjasteh A.H. (2026). Advances in immunotherapy for colorectal cancer: Overcoming resistance in mismatch repair-proficient tumors. Cancer Cell Int..

[14] Ji K., Jia H., Liu Z., Yu G., Wen R., Zhang T., Peng Z., Man W., Tian Y., Wang C. (2025). New insight in immunotherapy and combine therapy in colorectal cancer. Front. Cell Dev. Biol..

[15] Fadlallah H., El Masri J., Fakhereddine H., Youssef J., Chemaly C., Doughan S., Abou-Kheir W. (2024). Colorectal cancer: Recent advances in management and treatment. World J. Clin. Oncol..

[16] Wong S.H., Yu J. (2019). Gut microbiota in colorectal cancer: Mechanisms of action and clinical applications. Nat. Rev. Gastroenterol. Hepatol..

[17] Kostic A.D., Chun E., Robertson L., Glickman J.N., Gallini C.A., Michaud M., Clancy T.E., Chung D.C., Lochhead P., Hold G.L. (2013). Fusobacterium nucleatum potentiates intestinal tumorigenesis and modulates the tumor-immune microenvironment. Cell Host Microbe.

[18] Pleguezuelos-Manzano C., Puschhof J., Rosendahl Huber A., van Hoeck A., Wood H.M., Nomburg J., Gurjao C., Manders F., Dalmasso G., Stege P.B. (2020). Mutational signature in colorectal cancer caused by genotoxic pks^+^
*E. coli*. Nature.

[19] Helmink B.A., Khan M.A.W., Hermann A., Gopalakrishnan V., Wargo J.A. (2019). The microbiome, cancer, and cancer therapy. Nat. Med..

[20] Alexander J.L., Wilson I.D., Teare J., Marchesi J.R., Nicholson J.K., Kinross J.M. (2017). Gut microbiota modulation of chemotherapy efficacy and toxicity. Nat. Rev. Gastroenterol. Hepatol..

[21] Swanson K.S., Gibson G.R., Hutkins R., Reimer R.A., Reid G., Verbeke K., Scott K.P., Holscher H.D., Azad M.B., Delzenne N.M. (2020). The international scientific association for probiotics and prebiotics (ISAPP) consensus statement on the definition and scope of synbiotics. Nat. Rev. Gastroenterol. Hepatol..

[22] Louis P., Hold G.L., Flint H.J. (2014). The gut microbiota, bacterial metabolites and colorectal cancer. Nat. Rev. Microbiol..

[23] Janney A., Powrie F., Mann E.H. (2020). Host-microbiota maladaptation in colorectal cancer. Nature.

[24] Rafter J., Bennett M., Caderni G., Clune Y., Hughes R., Karlsson P.C., Klinder A., O’Riordan M., O’Sullivan G.C., Pool-Zobel B. (2007). Dietary synbiotics reduce cancer risk factors in polypectomized and colon cancer patients. Am. J. Clin. Nutr..

[25] Thursby E., Juge N. (2017). Introduction to the human gut microbiota. Biochem. J..

[26] Valdes A.M., Walter J., Segal E., Spector T.D. (2018). Role of the gut microbiota in nutrition and health. BMJ.

[27] Lloyd-Price J., Abu-Ali G., Huttenhower C. (2016). The healthy human microbiome. Genome Med..

[28] Zmora N., Suez J., Elinav E. (2019). You are what you eat: Diet, health and the gut microbiota. Nat. Rev. Gastroenterol. Hepatol..

[29] Rowland I., Gibson G., Heinken A., Scott K., Swann J., Thiele I., Tuohy K. (2018). Gut microbiota functions: Metabolism of nutrients and other food components. Eur. J. Nutr..

[30] Honda K., Littman D.R. (2016). The microbiota in adaptive immune homeostasis. Nature.

[31] Derrien M., Belzer C., de Vos W.M. (2017). Akkermansia muciniphila and its role in regulating host functions. Microb. Pathog..

[32] Qin J., Li R., Raes J., Arumugam M., Burgdorf K.S., Manichanh C., Nielsen T., Pons N., Levenez F., Yamada T. (2010). A human gut microbial gene catalogue established by metagenomic sequencing. Nature.

[33] Zoetendal E., Raes J., van den Bogert B., Arumugam M., Booijink C.C., Troost F.J., Bork P., Wels M., de Vos W.M., Kleerebezem M. (2012). The human small intestinal microbiota is driven by rapid uptake and conversion of simple carbohydrates. ISME J..

[34] Gaci N., Borrel G., Tottey W., O’Toole P.W., Brugère J.F. (2014). Archaea and the human gut: New beginning of an old story. World J. Gastroenterol..

[35] Shkoporov A.N., Hill C. (2019). Bacteriophages of the human gut: The “known unknown” of the microbiome. Cell Host Microbe.

[36] Richard M.L., Sokol H. (2019). The gut mycobiota: Insights into analysis, environmental interactions and role in gastrointestinal diseases. Nat. Rev. Gastroenterol. Hepatol..

[37] Human Microbiome Project Consortium (2012). Structure, function and diversity of the healthy human microbiome. Nature.

[38] Goodrich J.K., Waters J.L., Poole A.C., Sutter J.L., Koren O., Blekhman R., Beaumont M., Van Treuren W., Knight R., Bell J.T. (2014). Human genetics shape the gut microbiome. Cell.

[39] Singh G., Chaudhry Z., Boyadzhyan A., Sasaninia K., Rai V. (2025). Dysbiosis and colorectal cancer: Conducive factors, biological and molecular role, and therapeutic prospectives. Explor. Target. Anti-Tumor Ther..

[40] Karam F., El Deghel Y., Iratni R., Dakroub A.H., Eid A.H. (2025). The gut microbiome and colorectal cancer: An integrative review of the underlying mechanisms. Cell Biochem. Biophys..

[41] Grellier N., Severino A., Archilei S., Kim J., Gasbarrini A., Cammarota G., Porcari P., Benech N. (2024). Gut microbiota in inflammation and colorectal cancer: A potential toolbox for clinicians. Best Pract. Res. Clin. Gastroenterol..

[42] Zepeda-Rivera M., Minot S.S., Bouzek H., Wu H., Blanco-Míguez A., Manghi P., Jones D.S., LaCourse K.D., Wu Y., McMahon E.F. (2024). A distinct Fusobacterium nucleatum clade dominates the colorectal cancer niche. Nature.

[43] Ruiz-Malagón A.J., Rodríguez-Sojo M.J., Redondo E., Rodríguez-Cabezas M.E., Gálvez J., Rodríguez-Nogales A. (2025). Systematic review: The gut microbiota as a link between colorectal cancer and obesity. Obes. Rev..

[44] Rubinstein M.R., Wang X., Liu W., Hao Y., Cai G., Han Y.W. (2013). *Fusobacterium nucleatum* promotes colorectal carcinogenesis by modulating E-cadherin/β-catenin signaling. Cell Host Microbe.

[45] Gur C., Ibrahim Y., Isaacson B., Yamin R., Abed J., Gamliel M., Enk J., Bar-On Y., Stanietsky-Kaynan N., Coppenhagen-Glazer S. (2015). Binding of the Fap2 protein of *Fusobacterium nucleatum* to human inhibitory receptor TIGIT protects tumors from immune cell attack. Immunity.

[46] Wu S., Rhee K.J., Albesiano E., Rabizadeh S., Wu X., Yen H.R., Huso D.L., Brancati F.L., Wick E., McAllister F. (2009). A human colonic commensal promotes colon tumorigenesis via activation of T helper type 17 T cell responses. Nat. Med..

[47] Sears C.L., Garrett W.S. (2014). Microbes, microbiota, and colon cancer. Cell Host Microbe.

[48] Chung L., Thiele Orberg E., Geis A.L., Chan J.L., Fu K., DeStefano Shields C.E., Dejea C.M., Fathi P., Chen J., Finard B.B. (2018). Bacteroides fragilis toxin coordinates a pro-carcinogenic inflammatory cascade via targeting of colonic epithelial cells. Cell Host Microbe.

[49] Xia S., Ma L., Li H., Li Y., Yu L. (2025). Prevalence of enterotoxigenic *Bacteroides fragilis* in colorectal cancer: A systematic review and meta-analysis. Front. Cell. Infect. Microbiol..

[50] Sadeghi M., Mestivier D., Sobhani I. (2024). Contribution of pks^+^
*Escherichia coli* to colon carcinogenesis. Microorganisms.

[51] de Souza J.B., de Almeida Campos L.A., Palácio S.B., Brelaz-de-Castro M.C.A., Cavalcanti I.M.F. (2024). Prevalence and implications of pKs-positive *Escherichia coli* in colorectal cancer. Life Sci..

[52] Zhang G., Sun D. (2024). The synthesis of the novel *Escherichia coli* toxin-colibactin and its mechanisms of tumorigenesis of colorectal cancer. Front. Microbiol..

[53] Badero O.J., Ikedionwu O.I., Ajayi V., Jemiseye A.V. (2025). A review of susceptibility factors for colibactin-associated colorectal cancer in african populations. Cureus.

[54] Chen B., Ramazzotti D., Heide T., Spiteri I., Fernandez-Mateos J., James C., Magnani L., Graham T.A., Sottoriva A. (2023). Contribution of pks^+^
*E. coli* mutations to colorectal carcinogenesis. Nat. Commun..

[55] Jans M., Vereecke L. (2025). Physiological drivers of pks^+^
*E. coli* in colorectal cancer. Trends Microbiol..

[56] Long X., Wong C.C., Tong L., Chu E.S.H., Ho Szeto C., Go M.Y.Y., Coker O.O., Chan A.W.H., Chan F.K.L., Sung J.J.Y. (2019). Peptostreptococcus anaerobius promotes colorectal carcinogenesis and modulates tumour immunity. Nat. Microbiol..

[57] Bai B., Ma J., Xu W., Chen X., Chen X., Lv C., Su W., Li Y., Sun H., Zhang B. (2025). Gut microbiota and colorectal cancer: Mechanistic insights, diagnostic advances, and microbiome-based therapeutic strategies. Front. Microbiol..

[58] Dong X., Yang J., He L., Fang H., Wang L., Zhu J., Xu J., Song K., Xuan Z. (2026). The barrier–microbiota–inflammation axis in colorectal cancer: Mechanisms and emerging diagnostic & therapeutic strategies. Cancers.

[59] Bahrami A., Khalaji A., Bahri Najafi M., Sadati S., Raisi A., Abolhassani A., Eshraghi R., Khaksary Mahabady M., Rahimian N., Mirzaei H. (2024). NF-κB pathway and angiogenesis: Insights into colorectal cancer development and therapeutic targets. Eur. J. Med. Res..

[60] Sadati S., Khalaji A., Bonyad A., Khoshdooz S., Hosseini Kolbadi K.S., Bahrami A., Moeinfar M.S., Morshedi M., Ghamsaraian A., Eterafi M. (2025). NF-κB and apoptosis: Colorectal cancer progression and novel strategies for treatment. Eur. J. Med. Res..

[61] Kearns R. (2025). Gut modulation to regulate NF-κB in colorectal and gastric cancer therapy and inflammation. Cancer Immunol. Immunother..

[62] Wang Z., Chang Y., Sun H., Li Y., Tang T. (2024). Advances in molecular mechanisms of inflammatory bowel disease-associated colorectal cancer (Review). Oncol. Lett..

[63] Alhinai E.A., Walton G.E., Commane D.M. (2019). The role of the gut microbiota in colorectal cancer causation. Int. J. Mol. Sci..

[64] Pastorelli L., De Salvo C., Mercado J.R., Vecchi M., Pizarro T.T. (2013). Central role of the gut epithelial barrier in the pathogenesis of chronic intestinal inflammation: Lessons learned from animal models and human genetics. Front. Immunol..

[65] Ridlon J.M., Kang D.J., Hylemon P.B. (2006). Bile salt biotransformations by human intestinal bacteria. J. Lipid Res..

[66] Grivennikov S.I., Wang K., Mucida D., Stewart C.A., Schnabl B., Jauch D., Taniguchi K., Yu G.Y., Osterreicher C.H., Hung K.E. (2012). Adenoma-linked barrier defects and microbial products drive IL-23/IL-17-mediated tumour growth. Nature.

[67] Zitvogel L., Galluzzi L., Viaud S., Vétizou M., Daillère R., Merad M., Kroemer G. (2015). Cancer and the gut microbiota: An unexpected link. Sci. Transl. Med..

[68] Grivennikov S.I., Greten F.R., Karin M. (2010). Immunity, inflammation, and cancer. Cell.

[69] Hill C., Guarner F., Reid G., Gibson G.R., Merenstein D.J., Pot B., Morelli L., Canani R.B., Flint H.J., Salminen S. (2014). Expert consensus document. The international scientific association for probiotics and prebiotics consensus statement on the scope and appropriate use of the term probiotic. Nat. Rev. Gastroenterol. Hepatol..

[70] Markowiak P., Śliżewska K. (2017). Effects of probiotics, prebiotics, and synbiotics on human health. Nutrients.

[71] Smolinska S., Popescu F.D., Zemelka-Wiacek M. (2025). A review of the influence of prebiotics, probiotics, synbiotics, and postbiotics on the human gut microbiome and intestinal integrity. J. Clin. Med..

[72] Roberfroid M., Gibson G.R., Hoyles L., McCartney A.L., Rastall R., Rowland I., Wolvers D., Watzl B., Szajewska H., Stahl B. (2010). Prebiotic effects: Metabolic and health benefits. Br. J. Nutr..

[73] He Y., Peng K., Tan J., Hao Y., Zhang S., Gao C., Li L. (2025). Short-chain fatty acids and colorectal cancer: A systematic review and integrative bayesian meta-analysis of microbiome–metabolome interactions and intervention efficacy. Nutrients.

[74] Moreira M.M., Carriço M., Capelas M.L., Pimenta N., Santos T., Ganhão-Arranhado S., Mäkitie A., Ravasco P. (2024). The impact of pre-, pro- and synbiotics supplementation in colorectal cancer treatment: A systematic review. Front. Oncol..

[75] Kleerebezem M., Führen J. (2024). Synergistic *vs*. complementary synbiotics: The complexity of discriminating synbiotic concepts using a *Lactiplantibacillus plantarum* exemplary study. Microbiome Res. Rep..

[76] Al-Habsi N., Al-Khalili M., Haque S.A., Elias M., Olqi N.A., Al Uraimi T. (2024). health benefits of prebiotics, probiotics, synbiotics, and postbiotics. Nutrients.

[77] Cosier D.J., Lambert K., Neale E.P., Probst Y., Charlton K. (2025). The effect of oral synbiotics on the gut microbiota and inflammatory biomarkers in healthy adults: A systematic review and meta-analysis. Nutr. Rev..

[78] Gao Y., Yao Q., Meng L., Wang J., Zheng N. (2024). Double-side role of short chain fatty acids on host health via the gut-organ axes. Anim. Nutr..

[79] Parada Venegas D., De la Fuente M.K., Landskron G., González M.J., Quera R., Dijkstra G., Harmsen H.J.M., Faber K.N., Hermoso M.A. (2019). Short chain fatty acids (SCFAs)-mediated gut epithelial and immune regulation and its relevance for inflammatory bowel diseases. Front. Immunol..

[80] Koh A., De Vadder F., Kovatcheva-Datchary P., Bäckhed F. (2016). From dietary fiber to host physiology: Short-chain fatty acids as key bacterial metabolites. Cell.

[81] Fellows R., Denizot J., Stellato C., Cuomo A., Jain P., Stoyanova E., Balázsi S., Hajnády Z., Liebert A., Kazakevych J. (2018). Microbiota derived short chain fatty acids promote histone crotonylation in the colon through histone deacetylases. Nat. Commun..

[82] Canani R.B., Costanzo M.D., Leone L., Pedata M., Meli R., Calignano A. (2011). Potential beneficial effects of butyrate in intestinal and extraintestinal diseases. World J. Gastroenterol..

[83] Yu L.C. (2018). Microbiota dysbiosis and barrier dysfunction in inflammatory bowel disease and colorectal cancers: Exploring a common ground hypothesis. J. Biomed. Sci..

[84] Johnson D.E., O’Keefe R.A., Grandis J.R. (2018). Targeting the IL-6/JAK/STAT3 signalling axis in cancer. Nat. Rev. Clin. Oncol..

[85] Furusawa Y., Obata Y., Fukuda S., Endo T.A., Nakato G., Takahashi D., Nakanishi Y., Uetake C., Kato K., Kato T. (2013). Commensal microbe-derived butyrate induces the differentiation of colonic regulatory T cells. Nature.

[86] Tanoue T., Atarashi K., Honda K. (2016). Development and maintenance of intestinal regulatory T cells. Nat. Rev. Immunol..

[87] Kezer G., Paramithiotis S., Khwaldia K., Harahap I.A., Čagalj M., Šimat V., Smaoui S., Elfalleh W., Ozogul F., Esatbeyoglu T. (2025). A comprehensive overview of the effects of probiotics, prebiotics and synbiotics on the gut-brain axis. Front. Microbiol..

[88] Kan H.X., Cao Y., Ma Y., Zhang Y.L., Wang J., Li J., Li J.N. (2024). Efficacy and safety of probiotics, prebiotics, and synbiotics for the prevention of colorectal cancer and precancerous lesion in high-risk populations: A systematic review and meta-analysis of randomized controlled trials. J. Dig. Dis..

[89] Tegegne B.A., Abebaw D., Teffera Z.H., Fenta A., Belew H., Belayneh M., Jemal M., Getinet M., Baylie T., Tamene F.B. (2025). Microbial therapeutics in cancer: Translating probiotics, prebiotics, synbiotics, and postbiotics from mechanistic insights to clinical applications: A topical review. FASEB J..

[90] Yao B., Wei W., Zhang H. (2025). Efficacy of probiotics or synbiotics supplementation on chemotherapy-induced complications and gut microbiota dysbiosis in gastrointestinal cancer: A systematic review and meta-analysis. Eur. J. Clin. Nutr..

[91] Song M., Garrett W.S., Chan A.T. (2015). Nutrients, foods, and colorectal cancer prevention. Gastroenterology.

[92] Rinninella E., Raoul P., Cintoni M., Franceschi F., Miggiano G.A.D., Gasbarrini A., Mele M.C. (2019). What is the healthy gut microbiota composition? A changing ecosystem across age, environment, diet, and diseases. Microorganisms.

[93] Paduraru D.N., Palcau A.C., Dinca V.G., Ciuc D.M., Constantinescu A. (2025). The role of gut microbiota in colorectal cancer pathogenesis: A comprehensive literature review. Int. J. Mol. Sci..

[94] O’Keefe S.J. (2016). Diet, microorganisms and their metabolites, and colon cancer. Nat. Rev. Gastroenterol. Hepatol..

[95] Zitvogel L., Ma Y., Raoult D., Kroemer G., Gajewski T.F. (2018). The microbiome in cancer immunotherapy: Diagnostic tools and therapeutic strategies. Science.

[96] Gao S., Liao X., He Y., Yang J. (2025). Probiotics/synbiotics supplementation reduce the infection incidence in patients undergoing resection for colorectal cancer: An umbrella review. Front. Microbiol..

[97] Makki K., Deehan E.C., Walter J., Bäckhed F. (2018). the impact of dietary fiber on gut microbiota in host health and disease. Cell Host Microbe.

[98] Scott K.P., Gratz S.W., Sheridan P.O., Flint H.J., Duncan S.H. (2013). The influence of diet on the gut microbiota. Pharmacol. Res..

[99] Ríos-Covián D., Ruas-Madiedo P., Margolles A., Gueimonde M., de Los Reyes-Gavilán C.G., Salazar N. (2016). Intestinal short chain fatty acids and their link with diet and human health. Front. Microbiol..

[100] Roediger W.E. (1982). Utilization of nutrients by isolated epithelial cells of the rat colon. Gastroenterology.

[101] Donohoe D.R., Collins L.B., Wali A., Bigler R., Sun W., Bultman S.J. (2012). The Warburg effect dictates the mechanism of butyrate-mediated histone acetylation and cell proliferation. Mol. Cell.

[102] Waldecker M., Kautenburger T., Daumann H., Busch C., Schrenk D. (2008). Inhibition of histone-deacetylase activity by short-chain fatty acids and some polyphenol metabolites formed in the colon. J. Nutr. Biochem..

[103] Archer S.Y., Hodin R.A. (1999). Histone acetylation and cancer. Curr. Opin. Genet. Dev..

[104] Wilson A.J., Gibson P.R. (1997). Short-chain fatty acids promote the migration of colonic epithelial cells in vitro. Gastroenterology.

[105] Marks P.A., Xu W.S. (2009). Histone deacetylase inhibitors: Potential in cancer therapy. J. Cell. Biochem..

[106] Smith P.M., Howitt M.R., Panikov N., Michaud M., Gallini C.A., Bohlooly-Y M., Glickman J.N., Garrett W.S. (2013). The microbial metabolites, short-chain fatty acids, regulate colonic Treg cell homeostasis. Science.

[107] Peng L., Li Z.R., Green R.S., Holzman I.R., Lin J. (2009). Butyrate enhances the intestinal barrier by facilitating tight junction assembly via activation of AMP-activated protein kinase in Caco-2 cell monolayers. J. Nutr..

[108] Cani P.D., Amar J., Iglesias M.A., Poggi M., Knauf C., Bastelica D., Neyrinck A.M., Fava F., Tuohy K.M., Chabo C. (2007). Metabolic endotoxemia initiates obesity and insulin resistance. Diabetes.

[109] Fukata M., Chen A., Vamadevan A.S., Cohen J., Breglio K., Krishnareddy S., Hsu D., Xu R., Harpaz N., Dannenberg A.J. (2007). Toll-like receptor-4 promotes the development of colitis-associated colorectal tumors. Gastroenterology.

[110] Malki A., El Ruz R.A., Gupta I., Allouch A., Vranic S., Al Moustafa A.E. (2020). Molecular mechanisms of colon cancer progression and metastasis: Recent insights and advancements. Int. J. Mol. Sci..

[111] Hamer H.M., Jonkers D., Venema K., Vanhoutvin S., Troost F.J., Brummer R.J. (2008). Review article: The role of butyrate on colonic function. Aliment. Pharmacol. Ther..

[112] Donohoe D.R., Garge N., Zhang X., Sun W., O’Connell T.M., Bunger M.K., Bultman S.J. (2011). The microbiome and butyrate regulate energy metabolism and autophagy in the mammalian colon. Cell Metab..

[113] Tan J., McKenzie C., Potamitis M., Thorburn A.N., Mackay C.R., Macia L. (2014). The role of short-chain fatty acids in health and disease. Adv. Immunol..

[114] Greten F.R., Eckmann L., Greten T.F., Park J.M., Li Z.W., Egan L.J., Kagnoff M.F., Karin M. (2004). IKKbeta links inflammation and tumorigenesis in a mouse model of colitis-associated cancer. Cell.

[115] Azad M.A.K., Sarker M., Wan D. (2018). Immunomodulatory effects of probiotics on cytokine profiles. BioMed Res. Int..

[116] Plaza-Diaz J., Ruiz-Ojeda F.J., Gil-Campos M., Gil A. (2019). Mechanisms of action of probiotics. Adv. Nutr..

[117] Akira S., Takeda K. (2004). Toll-like receptor signalling. Nat. Rev. Immunol..

[118] Kawai T., Akira S. (2007). TLR signaling. Semin. Immunol..

[119] Hayden M.S., Ghosh S. (2004). Signaling to NF-kappaB. Genes. Dev..

[120] Oeckinghaus A., Hayden M.S., Ghosh S. (2011). Crosstalk in NF-κB signaling pathways. Nat. Immunol..

[121] Segain J.P., Raingeard de la Blétière D., Bourreille A., Leray V., Gervois N., Rosales C., Ferrier L., Bonnet C., Blottière H.M., Galmiche J.P. (2000). Butyrate inhibits inflammatory responses through NFkappaB inhibition: Implications for Crohn’s disease. Gut.

[122] Inan M.S., Rasoulpour R.J., Yin L., Hubbard A.K., Rosenberg D.W., Giardina C. (2000). The luminal short-chain fatty acid butyrate modulates NF-kappaB activity in a human colonic epithelial cell line. Gastroenterology.

[123] Davie J.R. (2003). Inhibition of histone deacetylase activity by butyrate. J. Nutr..

[124] Usami M., Kishimoto K., Ohata A., Miyoshi M., Aoyama M., Fueda Y., Kotani J. (2008). Butyrate and trichostatin A attenuate nuclear factor κB activation and tumor necrosis factor α secretion and increase prostaglandin E2 secretion in human epithelial cells. Nutr. Res..

[125] Baud V., Karin M. (2009). Is NF-kappaB a good target for cancer therapy? Hopes and pitfalls. Nat. Rev. Drug Discov..

[126] DiDonato J.A., Mercurio F., Karin M. (2012). NF-κB and the link between inflammation and cancer. Immunol. Rev..

[127] Song M., Chan A.T. (2019). Environmental factors, gut microbiota, and colorectal cancer prevention. Clin. Gastroenterol. Hepatol..

[128] Louis P., Flint H.J. (2017). Formation of propionate and butyrate by the human colonic microbiota. Environ. Microbiol..

[129] Ruemmele F.M., Schwartz S., Seidman E.G., Dionne S., Levy E., Lentze M.J. (2003). Butyrate induced Caco-2 cell apoptosis is mediated via the mitochondrial pathway. Gut.

[130] Chipuk J.E., Green D.R. (2008). How do BCL-2 proteins induce mitochondrial outer membrane permeabilization?. Trends Cell Biol..

[131] Ciulei G., Orasan O.H., Coste S.C., Cozma A., Negrean V., Procopciuc L.M. (2020). Vitamin D and the insulin-like growth factor system: Implications for colorectal neoplasia. Eur. J. Clin. Investig..

[132] Procopciuc L.M., Osian G. (2013). Lys751Gln XPD and Arg399Gln XRCC1 in Romanians. Association with sporadic colorectal cancer risk and different stages of carcinomas. Chirurgia.

[133] Osian G., Procopciuc L., Vlad L. (2007). MTHFR polymorphisms as prognostic factors in sporadic colorectal cancer. J. Gastrointest. Liver Dis..

[134] Tilg H., Adolph T.E., Gerner R.R., Moschen A.R. (2018). The intestinal microbiota in colorectal cancer. Cancer Cell.

[135] Klaunig J.E. (2018). Oxidative stress and cancer. Curr. Pharm. Des..

[136] Schwabe R.F., Jobin C. (2013). The microbiome and cancer. Nat. Rev. Cancer.

[137] Bischoff S.C., Barbara G., Buurman W., Ockhuizen T., Schulzke J.D., Serino M., Tilg H., Watson A., Wells J.M. (2014). Intestinal permeability—A new target for disease prevention and therapy. BMC Gastroenterol..

[138] Zhan T., Rindtorff N., Boutros M. (2017). Wnt signaling in cancer. Oncogene.

[139] Clevers H. (2006). Wnt/beta-catenin signaling in development and disease. Cell.

[140] Klaus A., Birchmeier W. (2008). Wnt signalling and its impact on development and cancer. Nat. Rev. Cancer.

[141] MacDonald B.T., Tamai K., He X. (2009). Wnt/beta-catenin signaling: Components, mechanisms, and diseases. Dev. Cell.

[142] Grivennikov S.I. (2013). Inflammation and colorectal cancer: Colitis-associated neoplasia. Semin. Immunopathol..

[143] Li Q., Geng S., Luo H., Wang W., Mo Y.Q., Luo Q., Wang L., Song G.B., Sheng J.P., Xu B. (2024). Signaling pathways involved in colorectal cancer: Pathogenesis and targeted therapy. Signal Transduct. Target. Ther..

[144] Thulasinathan B., Suvilesh K.N., Maram S., Grossmann E., Ghouri Y., Teixeiro E.P., Chan J., Kaif J.T., Rachagani S. (2025). The impact of gut microbial short-chain fatty acids on colorectal cancer development and prevention. Gut Microbes.

[145] Sun J., Chen S., Zang D., Sun H., Sun Y., Chen J. (2024). Butyrate as a promising therapeutic target in cancer: From pathogenesis to clinic (Review). Int. J. Oncol..

[146] Ben-Neriah Y., Karin M. (2011). Inflammation meets cancer, with NF-κB as the matchmaker. Nat. Immunol..

[147] Hoesel B., Schmid J.A. (2013). The complexity of NF-κB signaling in inflammation and cancer. Mol. Cancer.

[148] Naugler W.E., Karin M. (2008). NF-kappaB and cancer-identifying targets and mechanisms. Curr. Opin. Genet. Dev..

[149] Shin N.R., Whon T.W., Bae J.W. (2015). Proteobacteria: Microbial signature of dysbiosis in gut microbiota. Trends Biotechnol..

[150] Chang P.V., Hao L., Offermanns S., Medzhitov R. (2014). The microbial metabolite butyrate regulates intestinal macrophage function via histone deacetylase inhibition. Proc. Natl. Acad. Sci. USA.

[151] Vinolo M.A., Rodrigues H.G., Nachbar R.T., Curi R. (2011). Regulation of inflammation by short chain fatty acids. Nutrients.

[152] Morgan M.J., Liu Z.G. (2011). Crosstalk of reactive oxygen species and NF-κB signaling. Cell Res..

[153] Reuter S., Gupta S.C., Chaturvedi M.M., Aggarwal B.B. (2010). Oxidative stress, inflammation, and cancer: How are they linked?. Free Radic. Biol. Med..

[154] Zolfanelli C., Lauciello V., Di Ciancia A., Vagliasindi A., Varzakas T., D’Amore T. (2026). Short-chain fatty acids as functional postbiotics in colorectal cancer management. Biol. Life Sci. Forum.

[155] Dong Y., Zhang K., Wei J., Ding Y., Wang X., Hou H., Wu J., Liu T., Wang B., Cao H. (2023). Gut microbiota-derived short-chain fatty acids regulate gastrointestinal tumor immunity: A novel therapeutic strategy?. Front. Immunol..

[156] Porta C., Paglino C., Mosca A. (2014). Targeting PI3K/Akt/mTOR signaling in cancer. Front. Oncol..

[157] Hardie D.G., Ross F.A., Hawley S.A. (2012). AMPK: A nutrient and energy sensor that maintains energy homeostasis. Nat. Rev. Mol. Cell Biol..

[158] Schwitalla S., Ziegler P., Horst D. (2012). Loss of p53 in enterocytes generates an inflammatory microenvironment enabling invasion and lymph node metastasis of carcinogen-induced colorectal tumors. Cancer Cell.

[159] Fang D., Hawke D., Zheng Y., Xia Y., Meisenhelder J., Nika H., Mills G.B., Kobayashi R., Hunter T., Lu Z. (2007). Phosphorylation of beta-catenin by AKT promotes beta-catenin transcriptional activity. J. Biol. Chem..

[160] Singh N., Gurav A., Sivaprakasam S., Brady E., Padia R., Shi H., Thangaraju M., Prasad P.D., Manicassamy S., Munn D.H. (2014). Activation of Gpr109a, receptor for niacin and the commensal metabolite butyrate, suppresses colonic inflammation and carcinogenesis. Immunity.

[161] Fong W., Li Q., Yu J. (2020). Gut microbiota modulation: A novel strategy for prevention and treatment of colorectal cancer. Oncogene.

[162] Le Leu R.K., Hu Y., Brown I.L., Woodman R.J., Young G.P. (2010). Synbiotic intervention of Bifidobacterium lactis and resistant starch protects against colorectal cancer development in rats. Carcinogenesis.

[163] Uccello M., Malaguarnera G., Basile F., D’agata V., Malaguarnera M., Bertino G., Vacante M., Drago F., Biondi A. (2012). Potential role of probiotics on colorectal cancer prevention. BMC Surg..

[164] Thoda C., Touraki M. (2025). Molecular mechanisms of probiotic action against gastrointestinal cancers. Int. J. Mol. Sci..

[165] Leung H.K.M., Lo E.K.K., Chen C., Zhang F., Felicianna, Ismaiah M.J., El-Nezami H. (2025). Probiotic mixture attenuates colorectal tumorigenesis in murine AOM/DSS model by suppressing STAT3, inducing apoptotic p53 and modulating gut microbiota. Probiotics Antimicrob. Proteins.

[166] Shang F., Jiang X., Wang H., Guo S., Kang S., Xu B., Wang X., Chen S., Li N., Liu B. (2024). Bifidobacterium longum suppresses colorectal cancer through the modulation of intestinal microbes and immune function. Front. Microbiol..

[167] Zhang J., Zhang P., Li S., Yu T., Lai X., He Y. (2024). Study on the effect and mechanism of Lacticaseibacillus rhamnosus AFY06 on inflammation-associated colorectal cancer induced by AOM/DSS in mice. Front. Microbiol..

[168] Zhou T., Wu J., Khan A., Hu T., Wang Y., Salama E.S., Su S., Han H., Jin W., Li X. (2024). A probiotic Limosilactobacillus fermentum GR-3 mitigates colitis-associated tumorigenesis in mice via modulating gut microbiome. npj Sci. Food.

[169] Zhu J., Zheng X., Song X. (2026). Lactobacillus plantarum Zhang-LL alleviates colorectal cancer through the gut microbiome-arachidonic acid metabolism-intratumoral microbiota axis under antibiotic-driven intestinal dysbiosis. Food Biosci..

[170] Balkwill F.R., Mantovani A. (2012). Cancer-related inflammation: Common themes and therapeutic opportunities. Semin. Cancer Biol..

[171] Abdorrashidi M., Heiat M., Yeganeh A.V., Tohidinia A., Alizadeh A., Ramazani A., Gholizadeh H., Pouraskar T., Peypar M.H. (2025). *Lactobacillus casei*’s antitumor potential in colorectal cancer: Exploring mechanisms—A systematic review. BioMed Res. Int..

[172] Niechcial A., Schwarzfischer M., Wawrzyniak P., Determann M., Pöhlmann D., Wawrzyniak M., Gueguen E., Walker M.R., Morsy Y., Atrott K. (2025). Probiotic administration modulates gut microbiota and suppresses tumor growth in murine models of colorectal cancer. Int. J. Mol. Sci..

[173] Ma F., Sun M., Song Y., Wang A., Jiang S., Qian F., Mu G., Tuo Y. (2022). *Lactiplantibacillus plantarum*-12 alleviates inflammation and colon cancer symptoms in AOM/DSS-treated mice through modulating the intestinal microbiome and metabolome. Nutrients.

[174] Redman M.G., Ward E.J., Phillips R.S. (2014). The efficacy and safety of probiotics in people with cancer: A systematic review. Ann. Oncol..

[175] Chen C., Su Q., Zi M., Hua X., Zhang Z. (2025). Harnessing gut microbiota for colorectal cancer therapy: From clinical insights to therapeutic innovations. npj Biofilms Microbiomes.

[176] Yang Y., Xia Y., Chen H., Hong L., Feng J., Yang J., Yang Z., Shi C., Wu W., Gao R. (2016). The effect of perioperative probiotics treatment for colorectal cancer: Short-term outcomes of a randomized controlled trial. Oncotarget.

[177] Mego M., Holec V., Drgona L., Hainova K., Ciernikova S., Zajac V. (2013). Probiotic bacteria in cancer patients undergoing chemotherapy and radiation therapy. Complement. Ther. Med..

[178] Osterlund P., Ruotsalainen T., Korpela R., Saxelin M., Ollus A., Valta P., Kouri M., Elomaa I., Joensuu H. (2007). Lactobacillus supplementation for diarrhoea related to chemotherapy of colorectal cancer: A randomised study. Br. J. Cancer.

[179] Ding H., Zhang C., Yu L., Tian F., Chen W., Zhai Q. (2026). Probiotic or synbiotic interventions and chemotherapy-associated diarrhea in cancer patients: A meta-analysis of randomized trials. J. Future Foods.

[180] Meng S., Liu C., Zhang K., Li J., Wang D., Zhao J., Wang Y., Du M., Li C., Wang Y. (2025). A prebiotic-supplemented formula improves gut microbiota and intestinal inflammatory microenvironment in patients with colorectal adenoma: A double-blind, placebo-controlled trial. J. Nutr..

[181] Stene C., Xu J., Fallone de Andrade S., Palmquist I., Molin G., Ahrné S., Thorlacius H., Johnson L.B., Jeppsson B. (2025). Synbiotics protected radiation-induced tissue damage in rectal cancer patients: A controlled trial. Clin. Nutr..

[182] Zhang J.W., Du P., Gao J., Yang B.R., Fang W.J., Ying C.M. (2012). Preoperative probiotics decrease postoperative infectious complications of colorectal cancer. Am. J. Med. Sci..

[183] Kotzampassi K., Stavrou G., Damoraki G., Georgitsi M., Basdanis G., Tsaousi G., Giamarellos-Bourboulis E.J. (2015). A four-probiotics regimen reduces postoperative complications after colorectal surgery: A randomized, double-blind, placebo-controlled study. World J. Surg..

[184] Murovec B., Deutsch L., Stres B. (2024). Predictive modeling of colorectal cancer using exhaustive analysis of microbiome information layers available from public metagenomic data. Front. Microbiol..

[185] Negrut R.L., Cote A., Maghiar A.M. (2023). Exploring the Potential of Oral Microbiome Biomarkers for Colorectal Cancer Diagnosis and Prognosis: A Systematic Review. Microorganisms.

[186] Wang N., Fang J.Y. (2023). Fusobacterium nucleatum, a key pathogenic factor and microbial biomarker for colorectal cancer. Trends Microbiol..

[187] Li Z., Liu J., Li J., Zhou Z., Huang X., Gopinath D., Luo P., Wang Q., Shan D. (2025). Fusobacterium in the microbiome: From health to disease across the oral–gut axis and beyond. npj Biofilms Microbiomes.

[188] Zhang X., Chen Y., Xia Y., Lin S., Zhou X., Pang X., Yu J., Sun L. (2025). Oral microbiota in colorectal cancer: Unraveling mechanisms and application potential. Life Sci..

[189] Spanogiannopoulos P., Bess E.N., Carmody R.N., Turnbaugh P.J. (2016). The microbial pharmacists within us: A metagenomic view of xenobiotic metabolism. Nat. Rev. Microbiol..

[190] The Integrative HMP (iHMP) Research Network Consortium (2019). The integrative human microbiome project. Nature.

